# Variational Message Passing and Local Constraint Manipulation in Factor Graphs

**DOI:** 10.3390/e23070807

**Published:** 2021-06-24

**Authors:** İsmail Şenöz, Thijs van de Laar, Dmitry Bagaev, Bert de Vries

**Affiliations:** 1 Department of Electrical Engineering, Eindhoven University of Technology, 5600 MB Eindhoven, The Netherlands; T.W.v.d.Laar@tue.nl (T.v.d.L.); d.v.bagaev@tue.nl (D.B.); bert.de.vries@tue.nl (B.d.V.); 2 GN Hearing, JF Kennedylaan 2, 5612 AB Eindhoven, The Netherlands

**Keywords:** Bayesian inference, Bethe free energy, factor graphs, message passing, variational free energy, variational inference, variational message passing

## Abstract

Accurate evaluation of Bayesian model evidence for a given data set is a fundamental problem in model development. Since evidence evaluations are usually intractable, in practice variational free energy (VFE) minimization provides an attractive alternative, as the VFE is an upper bound on negative model log-evidence (NLE). In order to improve tractability of the VFE, it is common to manipulate the constraints in the search space for the posterior distribution of the latent variables. Unfortunately, constraint manipulation may also lead to a less accurate estimate of the NLE. Thus, constraint manipulation implies an engineering trade-off between tractability and accuracy of model evidence estimation. In this paper, we develop a unifying account of constraint manipulation for variational inference in models that can be represented by a (Forney-style) factor graph, for which we identify the Bethe Free Energy as an approximation to the VFE. We derive well-known message passing algorithms from first principles, as the result of minimizing the constrained Bethe Free Energy (BFE). The proposed method supports evaluation of the BFE in factor graphs for model scoring and development of new message passing-based inference algorithms that potentially improve evidence estimation accuracy.

## 1. Introduction

Building models from data is at the core of both science and engineering applications. The search for good models requires a performance measure that scores how well a particular model *m* captures the hidden patterns in a data set *D*. In a Bayesian framework, that measure is the *Bayesian evidence* p(D|m), i.e., the probability that model *m* would generate *D* if we were to draw data from *m*. The art of modeling is then the iterative process of proposing new model specifications, evaluating the evidence for each model and retaining the model with the most evidence [1].

Unfortunately, Bayesian evidence is intractable for most interesting models. A popular solution to evidence evaluation is provided by *variational* inference, which describes the process of Bayesian evidence evaluation as a (free energy) minimization process, since the variational free energy (VFE) is a tractable upper bound on Bayesian (negative log-)evidence [2]. In practice, the model development process then consists of proposing various candidate models, minimizing VFE for each model and selecting the model with the lowest minimized VFE.

The difference between VFE and negative log-evidence (NLE) is equal to the Kullback–Leibler divergence (KLD) [3] from the (perfect) Bayesian posterior distribution to the variational distribution for the latent variables in the model. The KLD can be interpreted as the cost of conducting variational rather than Bayesian inference. Perfect (Bayesian) inference would lead to zero inference costs (KLD =0), and the KLD increases as the variational posterior diverges further from the Bayesian posterior. As a result, model development in a variational inference context is a balancing act, where we search for models that have both large amounts of evidence for the data and small inference costs (small KLD). In other words, in a variational inference context, the researcher has two knobs to tune models. The first knob alters the model specification, which affects model evidence. The second knob relates to constraining the search space for the variational posterior, which may affect the inference costs.

In this paper, we are concerned with developing algorithms for tuning the second knob. How do we constrain the range of variational posteriors so as to make variational inferences both tractable and accurate (resulting in low KLD)? We present our framework in the context of a (Forney-style) factor graph representation of the model [4,5]. In that context, variational inference can be understood as an automatable and efficient message passing-based inference procedure [6,7,8].

Traditional constraints include mean-field [6] and Bethe approximations [9,10]. However, more recently it has become clear how alternative local constraints, such as posterior factorization [11], expectation and chance constraints [12,13], and local Laplace approximation [14], may impact both tractability and inference accuracy, and thereby potentially lead to lower VFE. The main contribution of the current work lies in unifying the various ideas on local posterior constraints into a principled method for deriving variational message passing-based inference algorithms. The proposed method derives existing message passing algorithms, but also supports the development of new message passing variants.

Section 2 reviews Forney-style Factor Graphs (FFGs) and variational inference by minimizing the Bethe Free Energy (BFE). This review is continued in Section 3, where we discuss BFE optimization from a Lagrangian optimization viewpoint. In Appendix A, we include an example to illustrate that the Bayes rule can be derived from Lagrangian optimization with data constraints. Our main contribution lies in Section 4, which provides a rigorous treatment of the effects of imposing local constraints on the BFE and the resulting message update rules. We build upon several previous works that describe how manipulation of (local) constraints and variational objectives can be employed to improve variational approximations in the context of message passing. For example, ref. [12] shows how inference algorithms can be unified in terms of hybrid message passing by Lagrangian constraint manipulation. We extend this view by bringing form (Section 4.2) and factorization constraints (Section 4.1) into a constrained optimization framework. In [15], a high-level recipe for generating message passing algorithms from divergence measures is described. We apply their general recipe in the current work, where we adhere to the view on local stationary points for region-based approximations on general graphs [16]. In Appendix B, we also show that locally stationary solutions are also the global stationary solutions. In Section 5, we develop an algorithm for VFE evaluation in an FFG. In previous work, ref. [17] describes a factor softening approach to evaluate the VFE for models with deterministic factors. We extend this work in Section 5, and show how to avoid factor softening for both free energy evaluation and inference of posteriors. We show an example of how to compute VFE for a deterministic node in Appendix C. A more detailed comparison to related work is given in Section 7.

In the literature, proofs and descriptions of message passing-based inference algorithms are scattered across multiple papers and varying graphical representations, including Bayesian networks [6,18], Markov random fields [16], bi-partite (Tanner) factor graphs [12,17,19] and Forney-style factor graphs (FFGs) [5,11]. In Appendix D, we provide first-principle proofs for a large collection of familiar message passing algorithms in the context of Forney-style factor graphs, which is the preferred framework in the information and communication theory communities [4,20].

## 2. Factor Graphs and the Bethe Free Energy

### 2.1. Terminated Forney-Style Factor Graphs

A Forney-style factor graph (FFG) is an undirected graph G=(V,E) with nodes V and edges E⊆V×V. We denote the neighboring edges of a node a∈V by E(a). Vice versa, for an edge i∈E, the notation V(i) collects all neighboring nodes. As a notational convention, we index nodes by a,b,c and edges by i,j,k, unless stated otherwise. We will mainly use *a* and *i* as summation indices and use the other indices to refer to a node or edge of interest.

In this paper, we will frequently refer to the notion of a subgraph. We define an edge-induced subgraph by G(i)=(V(i),i), and a node-induced subgraph by G(a)=(a,E(a)). Furthermore, we denote a local subgraph by G(a,i)=(V(i),E(a)), which collects all local nodes and edges around *i* and *a*, respectively.

An FFG can be used to represent a factorized function,

(1) $$\begin{array}{c} f\left(s\right)=\prod_{a\in V}f_{a}\left(s_{a}\right)\;, \end{array}$$

where $s_{a}$ collects the argument variables of factor $f_{a}$. We assumed that all the factors are positive. In an FFG, a node a∈V corresponds to a factor $f_{a}$, and the neighboring edges E(a) correspond to the variables $s_{a}$ that are the arguments of $f_{a}$.

**Figure 1:**
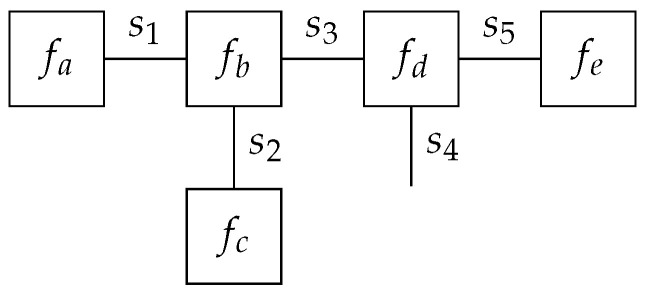
Example Forney-style factor graph for the model of (2).

As an example model, the following factorization (2), the corresponding FFG of which is shown in Figure 1.

(2) $$\begin{array}{c} f\left(s_{1},\cdots ,s_{5}\right)=f_{a}\left(s_{1}\right)\;f_{b}\left(s_{1},s_{2},s_{3}\right)\;f_{c}\left(s_{2}\right)\;f_{d}\left(s_{3},s_{4},s_{5}\right)\;f_{e}\left(s_{5}\right)\;. \end{array}$$

The FFG of Figure 1 consists of five nodes V={a,…,e}, as annotated by their corresponding factor functions, and five edges E={(a,b),…,(d,e)} as annotated by their corresponding variables. An edge that connects to only one node (e.g., the edge for $s_{4}$) is called a half-edge. In this example, the neighborhood E(b)={(a,b),(b,c),(b,d)} and V((b,c))={b,c}.

**Figure 2:**
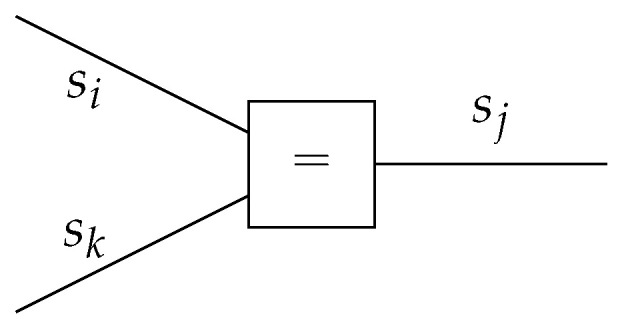
Visualization of the node-induced subgraph for an equality node. If the node function $f_{a}$ is known, a symbol representing the node function is often substituted within the node (“=” in this case).

In the FFG representation, a node can be connected to an arbitrary number of edges, while an edge can only be connected to at most two nodes. Therefore, FFGs often contain “equality nodes” that constrain connected edges to carry identical beliefs, with the implication that these beliefs can be made available to more than two factors. An equality node has the factor function

(3) $$\begin{array}{cc} f_{a}\left(s_{i},s_{j},s_{k}\right) & =\delta \left(s_{j}-s_{i}\right)\;\delta \left(s_{j}-s_{k}\right)\;, \end{array}$$

for which the node-induced subgraph G(a) is drawn in Figure 2.

If every edge in the FFG has exactly two connected nodes (including equality nodes), then we designate the graph as a terminated FFG (TFFG). Since multiplication of a function f(s) by 1 does not alter the function, any FFG can be terminated by connecting any half-edge *i* to a node *a* that represents the unity factor $f_{a}\left(s_{i}\right)=1$.

In Section 4.2 we discuss form constraints on posterior distributions. If such a constraint takes on a Dirac-delta functional form, then we visualize the constraint on the FFG by a small circle in the middle of the edge. For example, the small shaded circle in Figure 11 indicates that the variable has been observed. In Section 4.3.2 we consider form constraints in the context of optimization, in which case the circle annotation will be left open (see, e.g., Figure 14).

### 2.2. Variational Free Energy

Given a model f(s) and a (normalized) probability distribution q(s), we can define a Variational Free Energy (VFE) functional as

(4) $$F\left[q,f\right]≜\int q\left(s\right)\log\frac{q(s)}{f(s)}d\;s\;.$$

Variational inference is concerned with finding solutions to the minimization problem

(5) $$\begin{array}{c} q^{*}\;\left(s\right)=\arg\min_{q\in Q}F\left[q,f\right]\;, \end{array}$$

where Q imposes some constraints on *q*.

If *q* is unconstrained, then the optimal solution is obtained for $q^{*}\;\left(s\right)=p\left(s\right)$, with $p\left(s\right)=\frac{1}{Z}f\left(s\right)$ being the exact posterior, and Z=∫f(s)ds a normalizing constant that is commonly referred to as the evidence. The minimum value of the free energy then follows as the negative log-evidence (NLE),

$$\begin{array}{c} F[q^{*}\;,f]=-\log Z\;, \end{array}$$

which is also known as the surprisal. The NLE can be interpreted as a measure of model performance, where low NLE is preferred.

As an unconstrained search space for *q* grows exponentially with the number of variables, the optimization of (5) quickly becomes intractable beyond the most basic models. Therefore, constraints and approximations to the variational free energy (4) are often utilized. As a result, the *constrained* variational free energy with $q^{*}\in Q$ bounds the NLE by

(6) $$\begin{array}{c} F\left[q^{*}\;,f\right]=-\log Z+\int q^{*}\;\left(s\right)\log\frac{q^{*}\;\left(s\right)}{p(s)}d\;s\;, \end{array}$$

where the latter term expresses the divergence from the (intractable) exact solution to the optimal variational belief.

In practice, the functional form of q(s)=q(s;θ) is often parameterized, such that gradients of *F* can be derived w.r.t. the parameters θ. This effectively converts the variational optimization of F[q,f] to a parametric optimization of F(θ) as a function of θ. This problem can then be solved by a (stochastic) gradient descent procedure [21,22].

In the context of variational calculus, while form constraints may lead to interesting properties (see Section 4.2), they are generally not required. Interestingly, in a variational optimization context, the functional form of *q* is often not an *assumption*, but rather a *result* of optimization (see Section 4.3.1). An example of variational inference is provided in Appendix A.

### 2.3. Bethe Free Energy

The Bethe approximation enjoys a unique place in the landscape of Q, because the Bethe free energy (BFE) defines the fundamental objective of the celebrated belief propagation (BP) algorithm [17,23]. The origin of the Bethe approximation is rooted in tree-like approximations to subgraphs (possibly containing cycles) by enforcing local consistency conditions on the beliefs associated with edges and nodes [24].

Given a TFFG G=(V,E) for a factorized function $f\left(s\right)=\prod_{a\in V}f_{a}\left(s_{a}\right)$ (1), the Bethe free energy (BFE) is defined as [25]:

(7) $$\begin{array}{cc} F[q,f] & ≜\sum_{a\in V}\underset{F[q_{a},f_{a}]}{\underset{︸}{\int q_{a}\left(s_{a}\right)\log\frac{q_{a}\left(s_{a}\right)}{f_{a}\left(s_{a}\right)}d\;s_{a}}}+\sum_{i\in E}\underset{H[q_{i}]}{\underset{︸}{\int q_{i}\left(s_{i}\right)\log\frac{1}{q_{i}\left(s_{i}\right)}d\;s_{i}}} \end{array}$$

such that the factorized beliefs

(8) $$\begin{array}{cc} q(s) & =\prod_{a\in V}q_{a}\left(s_{a}\right)\prod_{i\in E}q_{i}{\left(s_{i}\right)}^{-1} \end{array}$$

satisfy the following constraints:

(9a) $$\begin{array}{cc} \int q_{a}\left(s_{a}\right)d\;s_{a} & =1\;,\;\mathrm{for}\;\mathrm{all}\;a\in V \end{array}$$

(9b) $$\begin{array}{cc} \int q_{a}\left(s_{a}\right)d\;s_{a\setminus i} & =q_{i}\left(s_{i}\right)\;,\;\mathrm{for}\;\mathrm{all}\;a\in V\;\mathrm{and}\;\mathrm{all}\;i\in E\left(a\right)\;. \end{array}$$

Together, the normalization constraint (9a) and marginalization constraint (9b) imply that the edge marginals are also normalized:

(10) $$\begin{array}{cc} \int q_{i}\left(s_{i}\right)d\;s_{i} & =1\;,\;\mathrm{for}\;\mathrm{all}\;i\in E\;. \end{array}$$

The Bethe free energy (7) includes a local free energy term $F[q_{a},f_{a}]$ for each node a∈V, and an entropy term $H[q_{i}]$ for each edge i∈E. Note that the local free energy also depends on the node function $f_{a}$, as specified in the factorization of *f* (1), whereas the entropy only depends on the local belief $q_{i}$.

The Bethe factorization (8) and constraints are summarized by the local polytope [26]

(11) $$\begin{array}{c} L\left(G\right)=\left\{q_{a}\;\mathrm{for}\;\mathrm{all}\;a\in V\;s.t.\;(9a),\;\mathrm{and}\;q_{i}\;\mathrm{for}\;\mathrm{all}\;i\in E\left(a\right)\;s.t.\;(9b)\right\}\;, \end{array}$$

which defines the constrained search space for the factorized variational distribution (8).

### 2.4. Problem Statement

In this paper, the problem is to find the beliefs in the local polytope that minimize the Bethe free energy

(12) $$\begin{array}{c} q^{*}\;\left(s\right)=\arg\min_{q\in L(G)}F\left[q,f\right]\;, \end{array}$$

where *q* is defined by (8), and where q∈L(G) offers a shorthand notation for optimizing over the individual beliefs in the local polytope. In the following sections, we will follow the Lagrangian optimization approach to derive various message passing-based inference algorithms.

### 2.5. Sketch of Solution Approach

The problem statement of Section 2.4 defines a global minimization of the beliefs in the Bethe factorization. Instead of solving the global optimization problem directly, we employ the factorization of the variational posterior and local polytope to subdivide the global problem statement in multiple *interdependent* local objectives.

From the BFE objective (12) and local polytope of (11), we can construct the Lagrangian

(13) $$\begin{array}{cc} L[q,f]= & \sum_{a\in V}F\left[q_{a},f_{a}\right]+\sum_{a\in V}\psi_{a}\left[\int q_{a}\left(s_{a}\right)d\;s_{a}-1\right]+\sum_{a\in V}\sum_{i\in E(a)}\int \lambda_{ia}\left(s_{i}\right)\left[q_{i}\left(s_{i}\right)-\int q_{a}\left(s_{a}\right)d\;s_{a\setminus i}\right]d\;s_{i}\\ \; & +\sum_{i\in E}H\left[q_{i}\right]+\sum_{i\in E}\psi_{i}\left[\int q_{i}\left(s_{i}\right)d\;s_{i}-1\right]\;, \end{array}$$

where the Lagrange multipliers $\psi_{a}$, $\psi_{i}$ and $\lambda_{ia}$ enforce the normalization and marginalization constraints of (9). It can be seen that this Lagrangian contains local beliefs $q_{a}$ and $q_{i}$, which are coupled through the $\lambda_{ia}$ Lagrange multipliers. The Lagrange multipliers $\lambda_{ia}$ are doubly indexed, because there is a multiplier associated with each marginalization constraint. The Lagrangian method then converts a constrained optimization problem of F[q,f] to an unconstrained optimization problem of L[q,f]. The total variation of the Lagrangian (13) can then be approached from the perspective of variations of the individual (coupled) local beliefs.

More specifically, given a locally connected pair b∈V,j∈E(b), we can rewrite the optimization of (12) in terms of the local beliefs $q_{b},q_{j}$, and the constraints in the local polytope

(14) $$\begin{array}{c} L\left(G\left(b,j\right)\right)=\left\{q_{b}\;s.t.\;(9a),\;\mathrm{and}\;q_{j}\;s.t.\;(9b)\right\}\;, \end{array}$$

that pertains to these beliefs. The problem then becomes finding local stationary solutions

(15) $$\begin{array}{cc} \left\{q_{b}^{*},q_{j}^{*}\right\} & =\arg\min_{L(G(b,j))}F\left[q,f\right]\;. \end{array}$$

Using (13), the optimization of (15) can then be written in the Lagrangian form

(16a) $$\begin{array}{cc} q_{b}^{*} & =\arg\min_{q_{b}}L_{b}\left[q_{b},f_{b}\right]\;, \end{array}$$

(16b) $$\begin{array}{cc} q_{j}^{*} & =\arg\min_{q_{j}}L_{j}\left[q_{j}\right]\;, \end{array}$$

where the Lagrangians $L_{b}$ and $L_{j}$ include the local polytope of (14) to rewrite (13) as an explicit functional of beliefs $q_{b}$ and $q_{j}$ (see, e.g., Lemmas 1 and 2). The combined stationary solutions to the local objectives then also comprise a stationary solution to the global objective (Appendix B).

The current paper shows how to identify stationary solutions to local objectives of the form (15), with the use of variational calculus, under varying constraints as imposed by the local polytope (14). Interestingly, the resulting fixed-point equations can be interpreted as message passing updates on the underlying TFFG representation of the model. In the following Section 3 and Section 4, we derive the local stationary solutions under a selection of constraints and show how these relate to known message passing update rules (Table 1). It then becomes possible to derive novel message updates and algorithms by simply altering the local polytope.

## 3. Bethe Lagrangian Optimization by Message Passing

### 3.1. Stationary Points of the Bethe Lagrangian

We wish to minimize the Bethe free energy under variations of the variational density. As the Bethe free energy factorizes over factors and variables (7), we first consider variations on separate node- and edge-induced subgraphs.

**Figure 3:**
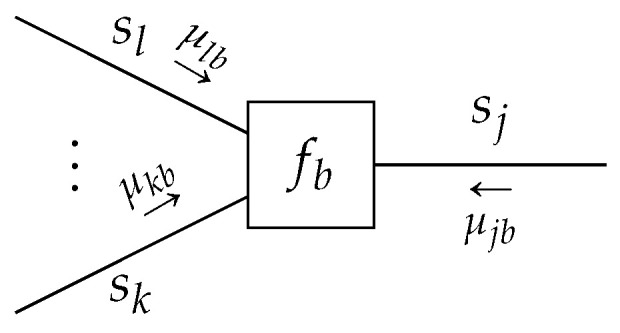
The subgraph around node *b* with indicated messages. Ellipses indicate an arbitrary (possibly zero) amount of edges.

**Lemma** **1.** *Given a TFFG*G=(V,E)*, consider the node-induced subgraph*G(b)*(Figure 3). The stationary points of the Lagrangian* (16a) *as a functional of* $q_{b}$*,*

(17) $$\begin{array}{cc} L_{b}\left[q_{b},f_{b}\right] & =F\left[q_{b},f_{b}\right]+\psi_{b}\left[\int q_{b}\left(s_{b}\right)d\;s_{b}-1\right]+\sum_{i\in E(b)}\int \lambda_{ib}\left(s_{i}\right)\left[q_{i}\left(s_{i}\right)-\int q_{b}\left(s_{b}\right)d\;s_{b\setminus i}\right]d\;s_{i}+C_{b}\;, \end{array}$$

*where $C_{b}$ collects all terms that are independent of $q_{b}$, which are of the form*

(18) $$q_{b}\left(s_{b}\right)=\frac{f_{b}\left(s_{b}\right)\prod_{i\in E(b)}\mu_{ib}\left(s_{i}\right)}{\int f_{b}\left(s_{b}\right)\prod_{i\in E(b)}\mu_{ib}\left(s_{i}\right)ds_{b}}\;.$$

**Proof.**  See Appendix D.1. □

The $\mu_{ib}\left(s_{i}\right)$ are any set of positive functions that makes (18) satisfy (9b), and will be identified in Theorem 1.

**Figure 4:**
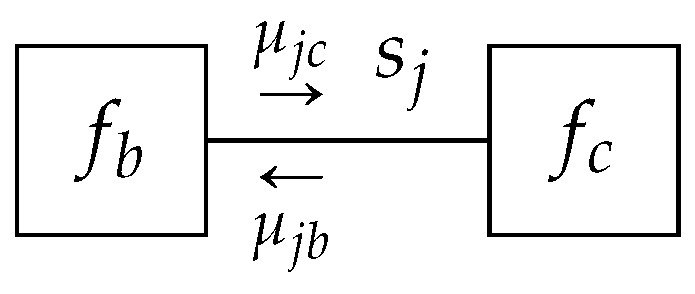
An edge-induced subgraph G(j) with indicated messages.

**Lemma** **2.** *Given a TFFG*G=(V,E)*, consider an edge-induced subgraph*G(j)*(Figure 4). The stationary points of the Lagrangian* (16b) *as a functional of* $q_{j}$*,*

(19) $$\begin{array}{cc} L_{j}\left[q_{j}\right] & =H\left[q_{j}\right]+\psi_{j}\left[\int q_{j}\left(s_{j}\right)d\;s_{j}-1\right]+\sum_{a\in V(j)}\int \lambda_{ja}\left(s_{j}\right)\left[q_{j}\left(s_{j}\right)-\int q_{a}\left(s_{a}\right)d\;s_{a\setminus j}\right]d\;s_{j}+C_{j}\;, \end{array}$$

*where* $C_{j}$ *collects all terms that are independent of* $q_{j}$*, are of the form*

(20) $$\begin{array}{cc} q_{j}\left(s_{j}\right) & =\frac{\mu_{jb}\left(s_{j}\right)\mu_{jc}\left(s_{j}\right)}{\int \mu_{jb}\left(s_{j}\right)\mu_{jc}\left(s_{j}\right)ds_{j}}\;. \end{array}$$

**Proof.**  See Appendix D.2. □

### 3.2. Minimizing the Bethe Free Energy by Belief Propagation

We now combine Lemmas 1 and 2 to derive the sum-product message update.

**Figure 5:**
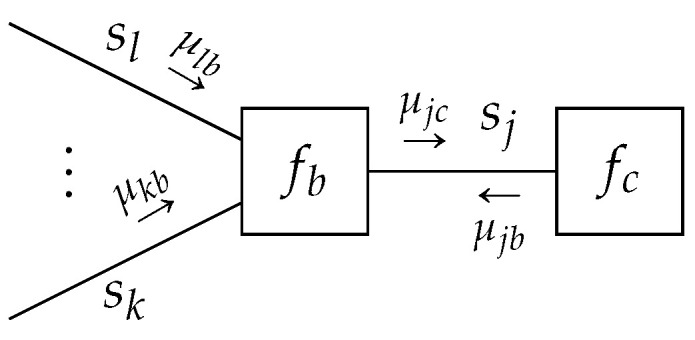
Visualization of a subgraph with indicated sum-product messages.

**Theorem** **1** (Sum-Product Message Update)**.** *Given a TFFG* G=(V,E)*, consider the induced subgraph* G(b,j) *(Figure 5). Given the local polytope* L(G(b,j)) *of* (14)*, then the local stationary solutions to* (15) *are given by*

(21a) $$\begin{array}{cc} q_{b}^{*}\left(s_{b}\right) & =\frac{f_{b}\left(s_{b}\right)\prod_{i\in E(b)}\mu_{ib}^{*}\left(s_{i}\right)}{\int f_{b}\left(s_{b}\right)\prod_{i\in E(b)}\mu_{ib}^{*}\left(s_{i}\right)ds_{b}} \end{array}$$

(21b) $$\begin{array}{cc} q_{j}^{*}\left(s_{j}\right) & =\frac{\mu_{jb}^{*}\left(s_{j}\right)\mu_{jc}^{*}\left(s_{j}\right)}{\int \mu_{jb}^{*}\left(s_{j}\right)\mu_{jc}^{*}\left(s_{j}\right)ds_{j}}\;, \end{array}$$

*with messages* $\mu_{jc}^{*}\left(s_{j}\right)$ *corresponding to the fixed points of*

(22) $$\begin{array}{cc} \mu_{jc}^{\left(k+1\right)}\left(s_{j}\right) & =\int f_{b}\left(s_{b}\right)\prod_{\begin{array}{c} i\in E(b)\\ i\neq j \end{array}}\mu_{ib}^{\left(k\right)}\left(s_{i}\right)ds_{b\setminus j}\;, \end{array}$$

*with k representing an iteration index.*

**Proof.**  See Appendix D.3. □

The sum-product algorithm has proven to be useful in many engineering applications and disciplines. For example, it is widely used for decoding in communication systems [4,20,27]. Furthermore, for a linear Gaussian state space model, Kalman filtering and smoothing can be expressed in terms of sum-product message passing for state inference on a factor graph [28,29]. This equivalence has inspired applications ranging from localization [30] to estimation [31].

The sum-product algorithm with updates (22) obtains the exact Bayesian posterior when the underlying graph is a tree [24,25,32]. Application of the sum-product algorithm to cyclic graphs is not guaranteed to converge and might lead to oscillations in the BFE over iterations. Theorems 3.1 and 3.2 in [33] show that the BFE of a graph with a single cycle is convex, which implies that the sum-product algorithm will converge in this case. Moreover, ref. [19] shows that it is possible to obtain a double-loop message passing algorithm if the graph has a cycle such that the stable fixed points will correspond to local minima of the BFE.

**Example** **1.** *A Linear Dynamical System Considering a Linear Gaussian state space model specified by the following factors:*

(23a) $$\begin{array}{cc} g_{0}\left(x_{0}\right) & =N(x_{0}|m_{x_{0}},V_{x_{0}}) \end{array}$$

(23b) $$\begin{array}{cc} g_{t}\left(x_{t-1},z_{t},A_{t}\right) & =\delta (z_{t}-A_{t}x_{t-1}) \end{array}$$

(23c) $$\begin{array}{cc} h_{t}\left(x_{t}^{\prime },z_{t},Q_{t}\right) & =N(x_{t}^{\prime }|z_{t},Q_{t}^{-1}) \end{array}$$

(23d) $$\begin{array}{cc} n_{t}\left(x_{t},x_{t}^{\prime },x_{t}^{\prime \prime }\right) & =\delta \left(x_{t}-x_{t}^{\prime }\right)\delta \left(x_{t}-x_{t}^{\prime \prime }\right) \end{array}$$

(23e) $$\begin{array}{cc} m_{t}\left(o_{t},x_{t}^{\prime \prime },B_{t}\right) & =\delta (o_{t}-B_{t}x_{t}^{\prime \prime }) \end{array}$$

(23f) $$\begin{array}{cc} r_{t}\left(y_{t},o_{t},R_{t}\right) & =N(y_{t}|o_{t},R_{t}^{-1})\;. \end{array}$$

**Figure 6:**
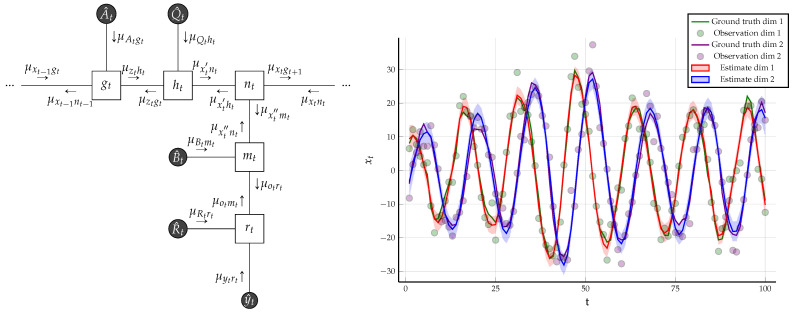
(**Left**) One time segment of the FFG corresponding to the linear Gaussian state space model specified in Example 1, with the sum-product messages computed according to (22). The three small dots at both sides of the graph indicate identical continuation of the graph over time. (**Right**) The small dots indicate the noisy observations that are synthetically generated by the linear state space model of (23) using parameter matrices as specified in (24). The posterior distribution for the hidden states are inferred by sum-product message passing and are drawn with shaded regions, indicating plus and minus the variance. The Bethe free energy evaluates to F[q,f]=580.698.

*The FFG corresponding to the one time segment of the state space model is given in Figure 6. We assumed that we know the following matrices that are used to generate the data:*

(24) $$\begin{array}{cc} {\hat{A}}_{t} & =\left[\begin{array}{cc} \cos(\theta ) & -\sin(\theta )\\ \sin(\theta ) & \cos(\theta ) \end{array}\right]\;,\;\;{\hat{Q}}_{t}^{-1}=\left[\begin{array}{cc} 3 & 0.1\\ 0.1 & 2 \end{array}\right]\;,\;\;{\hat{B}}_{t}=\left[\begin{array}{cc} 1 & 0\\ 0 & 1 \end{array}\right]\;,\;\;{\hat{R}}_{t}^{-1}=\left[\begin{array}{cc} 10 & 2\\ 2 & 20 \end{array}\right] \end{array}$$

*with* θ=π/8 *. Given a collection of observations* $\hat{y}=\left\{{\hat{y}}_{1},\ldots ,{\hat{y}}_{T}\right\}$ *, we constrain the latent states* $x=\{x_{0},\ldots ,x_{T}\}$ *by local marginalization and normalization constraints (for brevity we omit writing the normalization constraints explicitly) in accordance with Theorem 1, i.e.,*

(25a) $$\begin{array}{cc} \int q\left(x_{t-1},z_{t},A_{t}\right)dx_{t-1}dz_{t} & =q\left(A_{t}\right),\;\int q\left(x_{t-1},z_{t},A_{t}\right)dA_{t}=q\left(z_{t}|x_{t-1}\right)q\left(x_{t-1}\right) \end{array}$$

(25b) $$\begin{array}{cc} \int q\left(x_{t}^{\prime },z_{t},Q_{t}\right)dx_{t}^{\prime }dz_{t} & =q\left(Q_{t}\right),\;\int q\left(x_{t}^{\prime },z_{t},Q_{t}\right)dz_{t}dQ_{t}=q\left(x_{t}^{\prime }\right),\;\int q\left(x_{t}^{\prime },z_{t},Q_{t}\right)dx_{t}^{\prime }dQ_{t}=q\left(z_{t}\right) \end{array}$$

(25c) $$\begin{array}{cc} q(x_{t},x_{t}^{\prime },x_{t}^{\prime \prime }) & =q\left(x_{t}\right)\delta \left(x_{t}-x_{t}^{\prime }\right)\delta \left(x_{t}-x_{t}^{\prime \prime }\right) \end{array}$$

(25d) $$\begin{array}{cc} \int q\left(o_{t},x_{t}^{\prime \prime },B_{t}\right)do_{t},dx_{t}^{\prime \prime } & =q\left(B_{t}\right),\;\int q\left(o_{t},x_{t}^{\prime \prime },B_{t}\right)dB_{t}=q\left(o_{t}|x_{t}^{\prime \prime }\right)q\left(x_{t}^{\prime \prime }\right) \end{array}$$

(25e) $$\begin{array}{cc} \int q\left(o_{t},y_{t},R_{t}\right)do_{t}dy_{t} & =q\left(R_{t}\right),\;\int q\left(o_{t},y_{t},R_{t}\right)dR_{t}do_{t}=q\left(y_{t}\right),\;\int q\left(o_{t},y_{t},R_{t}\right)dR_{t}dy_{t}=q\left(o_{t}\right) \end{array}$$

*Moreover, we use data constraints in accordance with Theorem 3 (explained in Section 4.2.1) for the observations, state transition matrices and precision matrices, i.e.,*

$$\begin{array}{c} q\left(y_{t}\right)=\delta \left(y_{t}-{\hat{y}}_{t}\right),\;q\left(A_{t}\right)=\delta \left(A_{t}-{\hat{A}}_{t}\right),\;q\left(B_{t}\right)=\delta \left(B_{t}-{\hat{B}}_{t}\right),\;q\left(Q_{t}\right)=\delta \left(Q_{t}-{\hat{Q}}_{t}\right),\;q\left(R_{t}\right)=\delta \left(R_{t}-{\hat{R}}_{t}\right)\;. \end{array}$$

*Computation of sum-product messages by *(22)* is analytically tractable and detailed algebraic manipulation can be found in [31]. If the backwards messages are not passed, then the resulting sum-product message passing algorithm is equivalent to Kalman filtering and if both forward and backward messages are propagated, then the Rauch–Tung–Striebel smoother is obtained [34] (Ch. 8).*

*We generated*T=100*observations*$\hat{y}$*using the matrices specified in* (24) *and the initial condition* ${\hat{x}}_{0}={\left[5,-5\right]}^{⊤}$*. Due to* (23a)*, we have* $\mu_{x_{0}g_{1}}=N\left(m_{x_{0}},V_{x_{0}}\right)$*. We chose* $V_{x_{0}}=100\cdot I$ *and* $m_{x_{0}}={\hat{x}}_{0}$*. Under these constraints, the results of sum-product message passing and Bethe free energy evaluation is given in Figure 6. As the underlying graph is a tree, sum-product message passing results are exact and the evaluated BFE corresponds to negative log-evidence. In the follow-up Example 2, we will modify the constraints and give a comparative free energy plot for the examples in Figures 10 and 16.*

## 4. Message Passing Variations through Constraint Manipulation

For generic node functions with arbitrary connectivity, there is no guarantee that the sum-product updates can be solved analytically. When analytic solutions are not possible, there are two ways to proceed. One way is to try to solve the sum-product update equations numerically, e.g., by Monte Carlo methods. Alternatively, we can add additional constraints to the BFE that leads to simpler update equations at the cost of inference accuracy. In the remainder of the paper, we explore a variety of constraints that have proven to yield useful inference solutions.

### 4.1. Factorization Constraints

**Figure 7:**
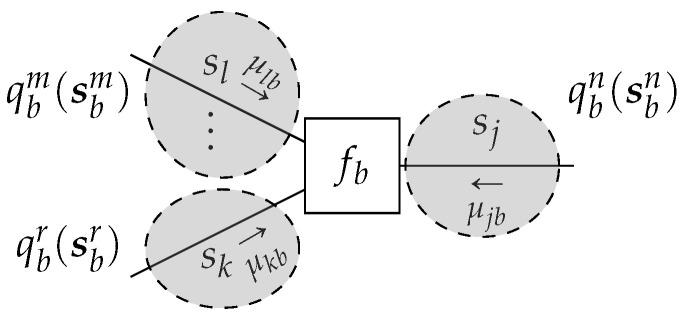
A node-induced subgraph G(b) with shaded sections that enclose the edges of an exemplary structured mean-field factorization l(b)={m,n,r}. Note that, in this example, the cluster *n* only encompasses the single edge *j*, such that $q_{b}^{n}\left(s_{b}^{n}\right)=q_{j}\left(s_{j}\right)$. In general, the assignment and number of edges in a cluster can be arbitrary.

Additional factorizations of the variational density $q_{a}\left(s_{a}\right)$ are often assumed to ease computation. In particular, we assumed a *structured mean-field factorization* such that

(26) $$q_{b}\left(s_{b}\right)≜\prod_{n\in l(b)}q_{b}^{n}\left(s_{b}^{n}\right)\;,$$

where *n* indicates a local cluster as a set of edges. To define a local cluster rigorously, let us first denote by P(a) the power set of an edge set E(a), where the power set is the set of all subsets of E(a). Then, a mean-field factorization l(a)⊆P(a) can be chosen such that all elements in E(a) are included in l(a) exactly once. Therefore, l(a) is defined as a set of one or multiple sets of edges. For example, if E(a)={i,j,k}, then l(a)={{i},{j,k}} is allowed, as is l(a)={{i,j,k}} itself, but l(a)={{i,j},{j,k}} is not allowed, since the element *j* occurs twice. More formally, in (26), the intersection of the super- and subscript collects the required variables, see Figure 7 for an example. The special case of a fully factorized l(b) for all edges i∈E(b) is known as the *naive mean-field factorization* [11,24].

We will analyze the effect of a structured mean-field factorization (26) on the Bethe free energy (7) for a specific factor node b∈V. Substituting (26) in the local free energy for factor *b* yields

(27) $$F\left[q_{b},f_{b}\right]=F\left[\left\{q_{b}^{n}\right\},f_{b}\right]=\sum_{n\in l(b)}\int q_{b}^{n}\left(s_{b}^{n}\right)\log q_{b}^{n}\left(s_{b}^{n}\right)d\;s_{b}^{n}-\int \left\{\prod_{n\in l(b)}q_{b}^{n}\left(s_{b}^{n}\right)\right\}\log f_{b}\left(s_{b}\right)d\;s_{b}\;.$$

We are then interested in

(28) $$\begin{array}{cc} q_{b}^{m,*} & =\arg\min_{q_{b}^{m}}L_{b}^{m}\left[q_{b}^{m},f_{b}\right]\;, \end{array}$$

where the Lagrangian $L_{b}^{m}$ (Lemma 3) enforces the normalization and marginalization constraints

(29a) $$\begin{array}{cc} \; & \int q_{b}^{m}\left(s_{b}^{m}\right)d\;s_{b}^{m}=1\;, \end{array}$$

(29b) $$\begin{array}{cc} \; & \int q_{b}^{m}\left(s_{b}^{m}\right)d\;s_{b\setminus i}^{m}=q_{i}\left(s_{i}\right),\;\mathrm{for}\;\mathrm{all}\;i\in m\;,m\in l\left(b\right)\;. \end{array}$$

**Lemma** **3.** *Given a terminated FFG* G=(V,E) *, consider a node-induced subgraph* G(b) *with a structured mean-field factorization* l(b) *(e.g., Figure 7). Then, local stationary solutions to the Lagrangian*

(30) $$\begin{array}{cc} L_{b}^{m}\left[q_{b}^{m}\right] & =\int q_{b}^{m}\left(s_{b}^{m}\right)\log q_{b}^{m}\left(s_{b}^{m}\right)d\;s_{b}^{m}-\int \left\{\prod_{n\in l(b)}q_{b}^{n}\left(s_{b}^{n}\right)\right\}\log f_{b}\left(s_{b}\right)d\;s_{b}+\\ \; & \;\psi_{b}^{m}\left[\int q_{b}^{m}\left(s_{b}^{m}\right)d\;s_{b}^{m}-1\right]+\sum_{i\in m}\int \lambda_{ib}\left(s_{i}\right)\left[q_{i}\left(s_{i}\right)-\int q_{b}^{m}\left(s_{b}^{m}\right)d\;s_{m\setminus i}\right]d\;s_{i}+C_{b}^{m}\;, \end{array}$$

*where* $C_{b}^{m}$ *collects all terms independent of* $q_{b}^{m}$ *, which are of the form*

(31) $$\begin{array}{cc} q_{b}^{m}\left(s_{b}^{m}\right) & =\frac{{\tilde{f}}_{b}^{m}\left(s_{b}^{m}\right)\prod_{i\in m}\mu_{ib}\left(s_{i}\right)}{\int {\tilde{f}}_{b}^{m}\left(s_{b}^{m}\right)\prod_{i\in m}\mu_{ib}\left(s_{i}\right)ds_{b}^{m}}\;, \end{array}$$

*where*

(32) $$\begin{array}{cc} {\tilde{f}}_{b}^{m}\left(s_{b}^{m}\right) & =\exp\left(\int \left\{\prod_{\begin{array}{c} n\in l(b)\\ n\neq m \end{array}}q_{b}^{n}\left(s_{b}^{n}\right)\right\}\log f_{b}\left(s_{b}\right)d\;s_{b}^{\setminus m}\right)\;. \end{array}$$

**Proof.**  See Appendix D.4. □

#### 4.1.1. Structured Variational Message Passing

We now combine Lemmas 2 and 3 to derive the structured variational message passing algorithm.

**Figure 8:**
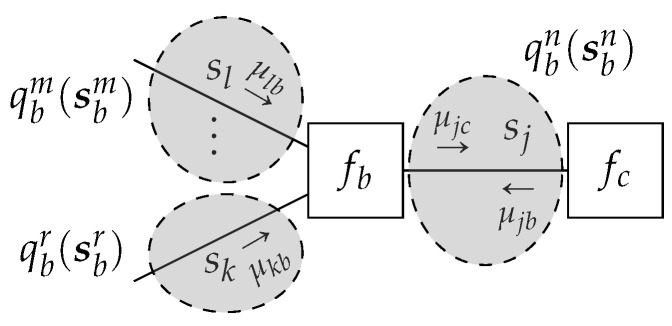
An example subgraph corresponding to G(b,j). Dashed ellipses enclose the edges of an exemplary exact cover l(b)={m,n,r}. In general, the assignment and number of edges in a cluster can be arbitrary.

**Theorem** **2.** *Structured variational message passing: Given a TFFG* G=(V,E) *, consider the induced subgraph* G(b,j) *with a structured mean-field factorization* l(b)⊆P(b) *, with local clusters* n∈l(b) *. Let* m∈l(b) *be the cluster where* j∈m *(see, e.g., Figure 8). Given the local polytope*

(33) $$\begin{array}{c} L\left(G\left(b,j\right)\right)=\left\{q_{b}^{n}\;for\;all\;n\in l\left(b\right)\;s.t.\;(29a),\;and\;q_{j}\;s.t.\;(29b)\right\}\;, \end{array}$$

*then local stationary solutions to*

(34) $$\begin{array}{cc} \left\{q_{b}^{m,*},q_{j}^{*}\right\} & =\arg\min_{L(G(b,j))}F\left[q,f\right]\;, \end{array}$$

*are given by*

(35a) $$\begin{array}{cc} q_{b}^{m,*}\left(s_{b}^{m}\right) & =\frac{{\tilde{f}}_{b}^{m,*}\left(s_{b}^{m}\right)\prod_{i\in m}\mu_{ib}^{*}\left(s_{i}\right)}{\int {\tilde{f}}_{b}^{m,*}\left(s_{b}^{m}\right)\prod_{i\in m}\mu_{ib}^{*}\left(s_{i}\right)ds_{b}^{m}} \end{array}$$

(35b) $$\begin{array}{cc} q_{j}^{*}\left(s_{j}\right) & =\frac{\mu_{jb}^{*}\left(s_{j}\right)\mu_{jc}^{*}\left(s_{j}\right)}{\int \mu_{jb}^{*}\left(s_{j}\right)\mu_{jc}^{*}\left(s_{j}\right)ds_{j}}\;, \end{array}$$

*with messages* $\mu_{jc}^{*}\left(s_{j}\right)$ *corresponding to the fixed points of*

(36) $$\begin{array}{cc} \mu_{jc}^{\left(k+1\right)}\left(s_{j}\right) & =\int {\tilde{f}}_{b}^{m,(k)}\left(s_{b}^{m}\right)\prod_{\begin{array}{c} i\in m\\ i\neq j \end{array}}\mu_{ib}^{\left(k\right)}\left(s_{i}\right)ds_{b\setminus j}^{m}\;, \end{array}$$

*with iteration index k, and where*

(37) $${\tilde{f}}_{b}^{m,(k)}=\exp\left(\int \left\{\prod_{\begin{array}{c} n\in l(b)\\ n\neq m \end{array}}q_{b}^{n,(k)}\left(s_{b}^{n}\right)\right\}\log f_{b}\left(s_{b}\right)d\;s_{b}^{\setminus m}\right)\;.$$

**Proof.**  See Appendix D.5. □

The structured mean-field factorization applies the marginalization constraint only to the local cluster beliefs, as opposed to the joint node belief. As a result, computation for the local cluster beliefs might become tractable [24] (Ch.5). The practical appeal of Variational Message Passing (VMP) based inference becomes evident when the underlying model is composed of conjugate factor pairs from the exponential family. When the underlying factors are conjugate exponential family distributions, the message passing updates (36) amounts to adding natural parameters [35] of the underlying exponential family distributions. Structured variational message passing is popular in acoustic signal modelling, e.g., [36], as it allows one to be able to keep track of correlations over time. In [37], a stochastic variant of structured variational inference is utilized for Latent Dirichlet Allocation. Structured approximations are also used to improve inference in auto-encoders. In [38], inference involving non-parametric Beta-Bernoulli process priors is improved by developing a structured approximation to variational auto-encoders. When the data being modelled are time series, structured approximations reflect the transition structure over time. In [39], an efficient structured black-box variational inference algorithm for fitting Gaussian variational models to latent time series is proposed.

**Example** **2.** *Consider the linear Gaussian state space model of Example 1. Let us assume that the precision matrix for latent-state transitions* $Q_{t}$ *is not known and can not be constrained by data. Then, we can augment state space model by including a prior for* $Q_{t}$ *and try to infer a posterior over* $Q_{t}$ *from the observations. Since* $Q_{t}$ *is the precision of a normal factor, we chose a conjugate Wishart prior and assumed that* $Q_{t}$ *is time-invariant by adding the following factors*

(38a) $$\begin{array}{cc} w_{0}\left(Q_{0},V,\nu \right) & =W(Q_{0}|V,\nu ) \end{array}$$

(38b) $$\begin{array}{cc} w_{t}\left(Q_{t-1},Q_{t},Q_{t+1}\right) & =\delta \left(Q_{t-1}-Q_{t}\right)\delta \left(Q_{t}-Q_{t+1}\right),\;for\;every\;t=1,\ldots ,T\;. \end{array}$$

*It is certainly possible to assume a time-varying structure for* $Q_{t}$ *; however, our purpose is to illustrate a change in constraints rather than analyzing time-varying properties. This is why we assume time-invariance.*

*In this setting, the sum-product equations around the factor*$h_{t}$*are not analytically tractable. Therefore, we changed the constraints associated with*$h_{t}$ (25b) *to those given in Theorem 2 as follows*

(39a) $$\begin{array}{cc} \int q\left(x_{t}^{\prime },z_{t},Q_{t}\right)dx_{t}^{\prime }dz_{t} & =q\left(Q_{t}\right),\;\int q\left(x_{t}^{\prime },z_{t},Q_{t}\right)dQ_{t}=q\left(x_{t}^{\prime },z_{t}\right) \end{array}$$

(39b) $$\begin{array}{cc} \int q\left(Q_{t}\right)dQ_{t} & =1,\;\int q\left(x_{t}^{\prime },z_{t}\right)dx_{t}^{\prime }dz_{t}=1\;. \end{array}$$

*We removed the data constraint on* $q(Q_{t})$ *and instead included data constraints on the hyper-parameters*

(40) $$\begin{array}{c} q\left(V\right)=\delta \left(V-\hat{V}\right),\;\;q\left(\nu \right)=\delta \left(\nu -\hat{\nu }\right)\;. \end{array}$$

**Figure 9:**
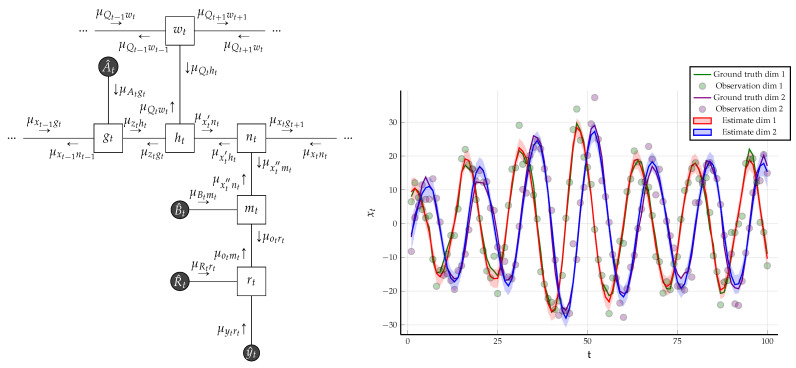
(**Left**) One time segment of the FFG corresponding to the linear Gaussian state space model specified in Example 2 with the sum-product messages computed according to (36). (**Right**) The small dots indicate the noisy observations that are synthetically generated by the linear state space model of (23) using matrices specified in (24). The posterior distribution of the hidden states inferred by structured variational message passing is depicted with shaded regions representing plus and minus one variances. The minimum of the evaluated Bethe free energy over all iterations is F[q,f]=586.178 (compared to F[q,f]=580.698 in Example 1). The posterior distribution for the precision matrix is given by $Q∼W\left(\left[\begin{array}{cc} 0.00266 & 0.000334\\ 0.00034 & 0.00670 \end{array}\right],102.0\right)$.

*With the new set of constraints ((39a) and (39b)), we obtained a hybrid of the sum-product and structured VMP algorithm, where structured messages around the factor* $h_{t}$ *are computed by* (36) *and the rest of the messages are computed by the sum-product* (22)*. One time segment of the modified FFG along with the messages is given Figure 9. We used the same observations* $\hat{y}$ *that were generated in Example 1 and the same initialization for the hidden states. For the hyper-parameters of the Wishart prior, we chose* $\hat{V}=0.1\cdot I$ *and* $\hat{\nu }=2$*. Under these constraints, the result of structured variational message passing results along with the Bethe free energy evaluation is given in Figure 9.*

#### 4.1.2. Naive Variational Message Passing

As a corollary of Theorem 2, we can consider the special case of a naive mean-field factorization, which is defined for node *b* as

(41) $$\begin{array}{c} q_{b}\left(s_{b}\right)=\prod_{i\in E(b)}q_{i}\left(s_{i}\right)\;. \end{array}$$

The naive mean-field constraint (41) transforms the local free energy into

(42) $$\begin{array}{cc} F[q_{b},f_{b}] & =F[\left\{q_{i}\right\},f_{b}]\\ \; & =\sum_{i\in E(b)}\int q_{i}\left(s_{i}\right)\log q_{i}\left(s_{i}\right)d\;s_{i}-\int \left\{\prod_{i\in E(b)}q_{i}\left(s_{i}\right)\right\}\log f_{b}\left(s_{b}\right)d\;s_{b}\;. \end{array}$$

**Corollary** **1.** *Naive Variational Message Passing: Given a TFFG G=(V,E), consider the induced subgraph G(b,j) with a naive mean-field factorization l(b)={isuchthatforalli∈E(b)}. Let m∈l(b) be the cluster where j=m. Given the local polytope of *(33)*, the local stationary solutions to *(34)* are given by*

$$\begin{array}{cc} q_{b}^{m,*}\left(s_{b}^{m}\right) & =q_{j}^{*}\left(s_{j}\right)=\frac{\mu_{jb}^{*}\left(s_{j}\right)\mu_{jc}^{*}\left(s_{j}\right)}{\int \mu_{jb}^{*}\left(s_{j}\right)\mu_{jc}^{*}\left(s_{j}\right)d\;s_{j}}\;, \end{array}$$

where the messages $\mu_{jc}^{*}\left(s_{j}\right)$ are the fixed points of the following iterations

(43) $$\begin{array}{cc} \mu_{jc}^{\left(k+1\right)}\left(s_{j}\right) & \;=\;\exp\left(\int \left\{\prod_{\begin{array}{c} i\in E(b)\\ i\neq j \end{array}}q_{i}^{\left(k\right)}\left(s_{i}\right)\right\}\log f_{b}\left(s_{b}\right)d\;s_{b\setminus j}\right)\;, \end{array}$$

where *k* is an iteration index.

**Proof.**  See Appendix D.6. □

The naive mean-field factorization limits the search space of beliefs by imposing strict constraints on the variational posterior. As a result, the variational posterior also loses flexibility. To improve inference performance for sparse Bayesian learning, the authors of [40] proposes a hybrid mechanism by augmenting naive mean-field VMP with sum-product updates. This hybrid scheme reduces the complexity of the sum-product algorithm, while improving the accuracy of the naive VMP approach. In [41], naive VMP is applied to semi-parametric regression and allows for scaling of regression models to large data sets.

**Example** **3.** *As a follow up on Example 2, we relaxed the constraints in ((39a) and (39b)) to the following constraints presented in Corollary 1 as*

(44a) $$\begin{array}{cc} \int q\left(x_{t}^{\prime },z_{t},Q_{t}\right)dx_{t}^{\prime }dz_{t} & =q\left(Q_{t}\right),\;\int q\left(x_{t}^{\prime },z_{t},Q_{t}\right)dQ_{t}=q\left(x_{t}^{\prime },z_{t}\right)=q\left(x_{t}^{\prime }\right)q\left(z_{t}\right) \end{array}$$

(44b) $$\begin{array}{cc} \int q\left(Q_{t}\right)dQ_{t} & =1,\;\int q\left(x_{t}^{\prime }\right)dx_{t}^{\prime }=1,\;\int q\left(z_{t}\right)dz_{t}=1\;. \end{array}$$

**Figure 10:**
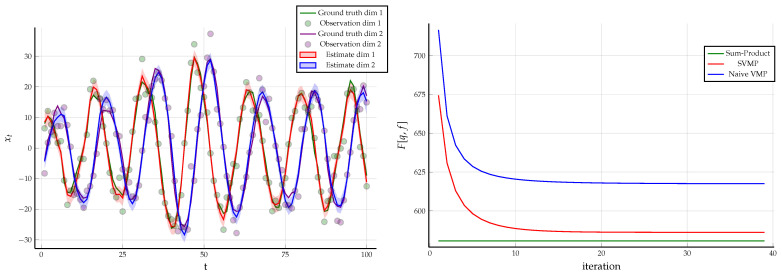
(**Left**) The small dots indicate the noisy observations that were synthetically generated by the linear state space model of (23) using matrices specified in (24). The posterior distribution for the hidden states inferred by naive variational message passing is depicted with shaded regions representing plus and minus one variances. The minimum of the evaluated Bethe free energy over all iterations is F[q,f]=617.468, which is more than for the less-constrained Example 2 (with F[q,f]=586.178) and Example 1 (with F[q,f]=580.698). The posterior for the precision matrix is given by $Q∼W\left(\left[\begin{array}{cc} 0.00141 & -6.00549e^{-5}\\ -6.00549e^{-5} & 0.00187 \end{array}\right],102.0\right)$. (**Right**) A comparison of the Bethe free energies for sum-product, structured and naive variational message passing algorithms for the data generated in Example 1.

*The FFG remains the same and we use identical data constraints as in Example 2. Together with constraint* (44)*, we obtained a hybrid of naive variational message passing and sum-product message passing algorithm where the messages around the factor* $h_{t}$ *are computed by* (43) *and the rest of the messages by sum-product* (22)*. Using the same data as in Example 1, the results for naive VMP are given in Figure 10 along with the evaluated Bethe free energy.*

### 4.2. Form Constraints

Form constraints limit the functional form of the variational factors $q_{a}\left(s_{a}\right)$ and $q_{i}\left(s_{i}\right)$. One of the most widely used form constraints, the data constraint, is also illustrated in Appendix A.

#### 4.2.1. Data Constraints

A data constraint can be viewed as a special case of (9b), where the belief $q_{j}$ is constrained to be a Dirac-delta function [42], such that

(45) $$\begin{array}{c} \int q_{a}\left(s_{a}\right)d\;s_{a\setminus j}=q_{j}\left(s_{j}\right)=\delta \left(s_{j}-{\hat{s}}_{j}\right)\;, \end{array}$$

where ${\hat{s}}_{j}$ is a known value, e.g., an observation.

**Lemma** **4.** *Given a TFFG* G=(V,E) *, consider the node-induced subgraph* G(b) *(Figure 3). Then local stationary solutions to the Lagrangian*

(46) $$\begin{array}{cc} L_{b}\left[q_{b},f_{b}\right] & =F\left[q_{b},f_{b}\right]+\psi_{b}\left[\int q_{b}\left(s_{b}\right)d\;s_{b}-1\right]+\sum_{\begin{array}{c} i\in E(b)\\ i\neq j \end{array}}\int \lambda_{ib}\left(s_{i}\right)\left[q_{i}\left(s_{i}\right)-\int q_{b}\left(s_{b}\right)d\;s_{b\setminus i}\right]d\;s_{i}+\\ \; & \;\int \lambda_{jb}\left(s_{j}\right)\left[\delta \left(s_{j}-{\hat{s}}_{j}\right)-\int q_{b}\left(s_{b}\right)d\;s_{b\setminus j}\right]d\;s_{j}+C_{b}\;. \end{array}$$

*where* $C_{b}$ *collects all terms that are independent of* $q_{b}$ *, are of the form*

(47) $$\begin{array}{cc} q_{b}\left(s_{b}\right) & =\frac{f_{b}\left(s_{b}\right)\prod_{i\in E(b)}\mu_{ib}\left(s_{i}\right)}{\int f_{b}\left(s_{b}\right)\prod_{i\in E(b)}\mu_{ib}\left(s_{i}\right)ds_{b}}\;. \end{array}$$

**Proof.**  See Appendix D.7. □

**Figure 11:**
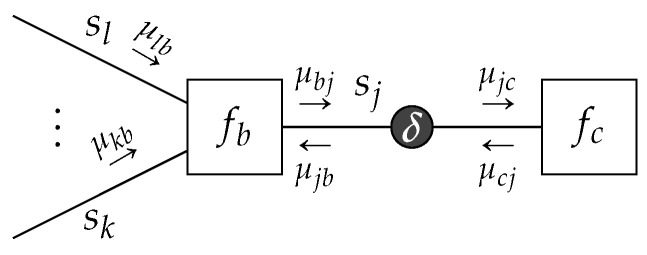
Visualization of a subgraph G(b,j) with indicated messages, where the dark circled delta indicates a data constraint—i.e., the variable $s_{j}$ is constrained to have a distribution of the form $\delta (s_{j}-{\hat{s}}_{j})$.

**Theorem** **3.** *Data-Constrained Sum-Product: Given a TFFG* G=(V,E) *, consider the induced subgraph* G(b,j) *(Figure 11). Given the local polytope*

(48) $$\begin{array}{c} L\left(G\left(b,j\right)\right)=\left\{q_{b}\;s.t.\;(45)\right\}\;, \end{array}$$

*the local stationary solutions to*

$$\begin{array}{c} q_{b}^{*}=\arg\min_{L(G(b,j))}F\left[q,f\right]\;, \end{array}$$

*are of the form*

(49) $$\begin{array}{cc} q_{b}^{*}\left(s_{b}\right) & =\frac{f_{b}\left(s_{b}\right)\prod_{i\in E(b)}\mu_{ib}^{*}\left(s_{i}\right)}{\int f_{b}\left(s_{b}\right)\prod_{i\in E(b)}\mu_{ib}^{*}\left(s_{i}\right)ds_{b}}\;, \end{array}$$

*with message*

(50) $$\begin{array}{cc} \mu_{jb}^{*}\left(s_{j}\right) & =\delta (s_{j}-{\hat{s}}_{j})\;. \end{array}$$

**Proof.**  See Appendix D.8. □

Note that the resulting message $\mu_{jb}^{*}\left(s_{j}\right)$ to node *b* does not depend on messages from node *c*, as would be the case for a sum-product update. By the symmetry of Theorem 3 for the subgraph L{G(c,j)}, (A32) identifies

$$\begin{array}{c} \mu_{cj}\left(s_{j}\right)=\int f_{c}\left(s_{c}\right)\prod_{\begin{array}{c} i\in E(c)\\ i\neq j \end{array}}\mu_{ic}\left(s_{i}\right)d\;s_{c\setminus j}\neq \delta \left(s_{j}-{\hat{s}}_{j}\right)\;. \end{array}$$

This implies that messages incoming to a data constraint (such as $\mu_{cj}$) are not further propagated through the data constraint. The data constraint thus effectively introduces a conditional independence between the variables of neighboring factors (conditioned on the shared constrained variable). Interestingly, this is similar to the notion of an intervention [43], where a decision variable is externally forced to a realization.

Data constraints allow information from data sets to be absorbed into the model. Essentially, (variational) Bayesian machine learning is an application of inference in a graph with data constraints. In our framework, data are a constraint, and machine learning via Bayes rule follows naturally from the minimization of the Bethe free energy (see also Appendix A).

#### 4.2.2. Laplace Propagation

A second type of form constraint we consider is the Laplace constraint, see also [14]. Consider a second-order Taylor approximation on the local log-node function

(51) $$\begin{array}{c} L_{a}\left(s_{a}\right)=\log f_{a}\left(s_{a}\right)\;, \end{array}$$

around an approximation point ${\hat{s}}_{a}$, as

(52) $$\begin{array}{c} {\tilde{L}}_{a}\left(s_{a};{\hat{s}}_{a}\right)=L_{a}\left({\hat{s}}_{a}\right)+\nabla^{⊤}\;L_{a}\left({\hat{s}}_{a}\right)\left(s_{a}-{\hat{s}}_{a}\right)+\frac{1}{2}{\left(s_{a}-{\hat{s}}_{a}\right)}^{⊤}\;\nabla^{2}L_{a}\left({\hat{s}}_{a}\right)\left(s_{a}-{\hat{s}}_{a}\right)\;. \end{array}$$

From this approximation, we define the Laplace-approximated node function as

(53) $$\begin{array}{cc} {\tilde{f}}_{a}\left(s_{a};{\hat{s}}_{a}\right) & ≜\exp\left({\tilde{L}}_{a}\left(s_{a};{\hat{s}}_{a}\right)\right)\;, \end{array}$$

which is substituted in the local free energy to obtain the Laplace-encoded local free energy as

(54) $$\begin{array}{cc} F[q_{a},{\tilde{f}}_{a};{\hat{s}}_{a}] & =\int q_{a}\left(s_{a}\right)\log\frac{q_{a}\left(s_{a}\right)}{{\tilde{f}}_{a}\left(s_{a};{\hat{s}}_{a}\right)}d\;s_{a}\;. \end{array}$$

It follows that the Laplace-encoded optimization of the local free energy becomes

(55) $$\begin{array}{cc} q_{a}^{*} & =\arg\min_{q_{a}}L_{a}\left[q_{a},{\tilde{f}}_{a};{\hat{s}}_{a}\right]\;, \end{array}$$

where the Lagrangian $L_{a}$ imposes the marginalization and normalization constraints of (9) on (54).

**Figure 12:**
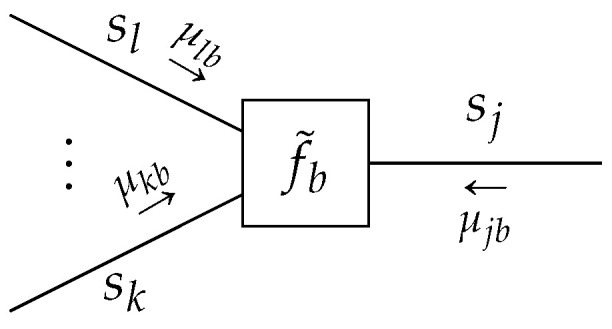
The subgraph around a Laplace-approximated node *b* with indicated messages.

**Lemma** **5.** *Given a TFFG*G=(V,E)*, consider the node-induced subgraph*G(b)*(Figure 12). The stationary points of the Laplace-approximated Lagrangian* (55) *as a functional of* $q_{b}$*,*

(56) $$\begin{array}{cc} L_{b}\left[q_{b},{\tilde{f}}_{b};{\hat{s}}_{b}\right] & =F\left[q_{b},{\tilde{f}}_{b};{\hat{s}}_{b}\right]+\psi_{b}\left[\int q_{b}\left(s_{b}\right)d\;s_{b}-1\right]+\\ \; & \;\sum_{i\in E(b)}\int \lambda_{ib}\left(s_{i}\right)\left[q_{i}\left(s_{i}\right)-\int q_{b}\left(s_{b}\right)d\;s_{b\setminus i}\right]d\;s_{i}+C_{b}\;, \end{array}$$

*where* $C_{b}$ *collects all terms that are independent of* $q_{b}$*, which are of the form*

(57) $$q_{b}\left(s_{b}\right)=\frac{{\tilde{f}}_{b}\left(s_{b};{\hat{s}}_{b}\right)\prod_{i\in E(b)}\mu_{ib}\left(s_{i}\right)}{\int {\tilde{f}}_{b}\left(s_{b};{\hat{s}}_{b}\right)\prod_{i\in E(b)}\mu_{ib}\left(s_{i}\right)ds_{b}}\;.$$

**Proof.**  See Appendix D.9. □

We can now formulate Laplace propagation as an iterative procedure, where the approximation point ${\hat{s}}_{b}$ is chosen as the mode of the belief $q_{b}\left(s_{b}\right)$.

**Figure 13:**
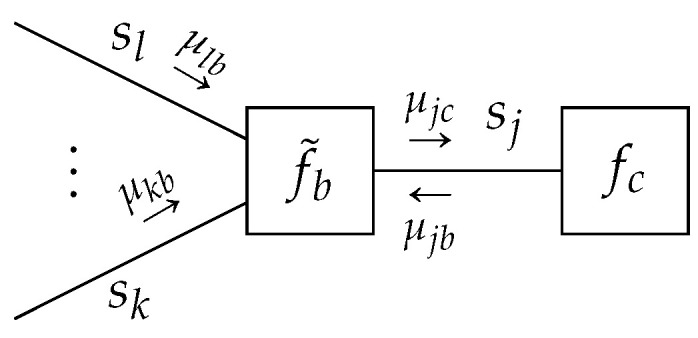
Visualization of a subgraph with indicated Laplace propagation messages. The node function $f_{b}$ is denoted by ${\tilde{f}}_{b}$ according to (53).

**Theorem** **4.** *
Laplace Propagation: Given a TFFG*G=(V,E)*, consider the induced subgraph*G(b,j)*(Figure 13) with the Laplace-encoded factor*${\tilde{f}}_{b}$*as per* (53)*. We write the model* (1) *with the Laplace-encoded factor* ${\tilde{f}}_{b}$ *substituted for* $f_{b}$*, as* $\tilde{f}$*. Given the local polytope* L(G(b,j)) *of* (14)*, the local stationary solutions to*

(58) $$\begin{array}{cc} \left\{q_{b}^{*},q_{j}^{*}\right\} & =\arg\min_{L(G(b,j))}F\left[q,\tilde{f};{\hat{s}}_{b}\right]\;, \end{array}$$

*are given by*

$$\begin{array}{cc} q_{b}^{*}\left(s_{b}\right) & =\frac{{\tilde{f}}_{b}\left(s_{b};{\hat{s}}_{b}^{*}\right)\prod_{i\in E(b)}\mu_{ib}^{*}\left(s_{i}\right)}{\int {\tilde{f}}_{b}\left(s_{b};{\hat{s}}_{b}^{*}\right)\prod_{i\in E(b)}\mu_{ib}^{*}\left(s_{i}\right)ds_{b}}\\ q_{j}^{*}\left(s_{j}\right) & =\frac{\mu_{jb}^{*}\left(s_{j}\right)\mu_{jc}^{*}\left(s_{j}\right)}{\int \mu_{jb}^{*}\left(s_{j}\right)\mu_{jc}^{*}\left(s_{j}\right)ds_{j}}\;, \end{array}$$

with ${\hat{s}}_{b}^{*}$ and the messages $\mu_{jc}^{*}\left(s_{j}\right)$ the fixed points of

$$\begin{array}{cc} {\hat{s}}_{b}^{\left(k\right)} & =\arg\max_{s_{b}}\;\log q_{b}^{\left(k\right)}\left(s_{b}\right)\\ q_{b}^{\left(k+1\right)}\left(s_{b}\right) & =\frac{{\tilde{f}}_{b}\left(s_{b};{\hat{s}}_{b}^{\left(k\right)}\right)\prod_{i\in E(b)}\mu_{ib}^{\left(k\right)}\left(s_{i}\right)}{\int {\tilde{f}}_{b}\left(s_{b};{\hat{s}}_{b}^{\left(k\right)}\right)\prod_{i\in E(b)}\mu_{ib}^{\left(k\right)}\left(s_{i}\right)ds_{b}}\\ \mu_{jc}^{\left(k+1\right)}\left(s_{j}\right) & =\int {\tilde{f}}_{b}\left(s_{b};{\hat{s}}_{b}^{\left(k\right)}\right)\prod_{\begin{array}{c} i\in E(b)\\ i\neq j \end{array}}\mu_{ib}^{\left(k\right)}\left(s_{i}\right)ds_{b\setminus j}\;. \end{array}$$

**Proof.**  See Appendix D.10. □

A Laplace propagation is introduced in [14] as an algorithm that propagates mean and variance information when exact updates are expensive to compute. Laplace propagation has found applications in the context of Gaussian processes and support vector machines [14]. In the jointly normal case, Laplace propagation coincides with sum-product and expectation propagation [14,18].

#### 4.2.3. Expectation Propagation

Expectation propagation can be derived in terms of constraint manipulation by relaxing the marginalization constraints to expectation constraints. Expectation constraints are of the form

(59) $$\begin{array}{c} \int q_{a}\left(s_{a}\right)T_{i}\left(s_{i}\right)ds_{a}=\int q_{i}\left(s_{i}\right)T_{i}\left(s_{i}\right)ds_{i}\;, \end{array}$$

for a given function (statistic) $T_{i}\left(s_{i}\right)$. Technically, the statistic $T_{i}\left(s_{i}\right)$ can be chosen arbitrarily. Nevertheless, they are often chosen as sufficient statistics of an exponential family distribution. An exponential family distribution is defined by

(60) $$q_{i}\left(s_{i}\right)=h\left(s_{i}\right)\exp\left(\eta_{i}^{⊤}T_{i}\left(s_{i}\right)-\log Z\left(\eta_{i}\right)\right)\;,$$

where $\eta_{i}$ is the natural parameter, $Z(\eta_{i})$ is the partition function, $T_{i}\left(s_{i}\right)$ is the sufficient statistics and $h(s_{i})$ is a base measure [24]. The reason $T_{i}\left(s_{i}\right)$ is a sufficient statistic is because if there are observed values of the random variable $s_{i}$, then the parameter $\eta_{i}$ can be estimated by using only the statistics $T_{i}\left(s_{i}\right)$. This means that the estimator of $\eta_{i}$ will depend only on the statistics.

The idea behind expectation propagation [18] is to relax the marginalization constraints with moment-matching constraints by choosing sufficient statistics from exponential family distributions [12]. Relaxation allows approximating the marginals of the sum-product algorithm with exponential family distributions. By keeping the marginals within the exponential family, the complexity of the resulting computations is reduced.

**Lemma** **6.** *Given a TFFG* G=(V,E) *, consider the node-induced subgraph* G(b) *(Figure 3). The stationary points of the Lagrangian*

$$\begin{array}{cc} L_{b}\left[q_{b},f_{b}\right] & =F\left[q_{b},f_{b}\right]+\psi_{b}\left[\int q_{b}\left(s_{b}\right)d\;s_{b}-1\right]+\sum_{\begin{array}{c} i\in E(b)\\ i\neq j \end{array}}\int \lambda_{ib}\left(s_{i}\right)\left[q_{i}\left(s_{i}\right)-\int q_{b}\left(s_{b}\right)d\;s_{b\setminus i}\right]d\;s_{i}+ \end{array}$$

(61) $$\begin{array}{c} \;\eta_{jb}^{⊤}\left[\int q_{j}\left(s_{j}\right)T_{j}\left(s_{j}\right)ds_{j}-\int q_{b}\left(s_{b}\right)T_{j}\left(s_{j}\right)ds_{b}\right]+C_{b}\;, \end{array}$$

*with sufficient statistics* $T_{j}$ *, and where* $C_{b}$ *collects all terms that are independent of* $q_{b}$ *, are of the form*

(62) $$\begin{array}{cc} q_{b}\left(s_{b}\right) & =\frac{f_{b}\left(s_{b}\right)\prod_{i\in E(b)}\mu_{ib}\left(s_{i}\right)}{\int f_{b}\left(s_{b}\right)\prod_{i\in E(b)}\mu_{ib}\left(s_{i}\right)ds_{b}}\;, \end{array}$$

*with incoming exponential family message*

(63) $$\begin{array}{cc} \mu_{jb}\left(s_{j}\right) & \;=\;\exp\;\left(\eta_{jb}^{⊤}T_{j}\left(s_{j}\right)\right)\;. \end{array}$$

**Proof.**  See Appendix D.11. □

**Lemma** **7.** *
Given a TFFG G=(V,E), consider an edge-induced subgraph G(j) (Figure 4). The stationary solutions of the Lagrangian*

$$\begin{array}{cc} L_{j}\left[q_{j}\right] & =H\left[q_{j}\right]+\psi_{j}\left[\int q_{j}\left(s_{j}\right)d\;s_{j}-1\right]+\sum_{a\in V(j)}\eta_{ja}^{⊤}\left[\int q_{j}\left(s_{j}\right)T_{j}\left(s_{j}\right)ds_{j}-\int q_{a}\left(s_{a}\right)T_{j}\left(s_{j}\right)ds_{a}\right]+C_{j}\;, \end{array}$$

*with sufficient statistics $T_{j}\left(s_{j}\right)$, and where $C_{j}$ collects all terms that are independent of $q_{j}$, are of the form*

(64) $$\begin{array}{cc} q_{j}\left(s_{j}\right) & =\frac{\exp\;\left({\left[\eta_{jb}+\eta_{jc}\right]}^{⊤}T_{j}\left(s_{j}\right)\right)}{\int \exp\;\left({\left[\eta_{jb}+\eta_{jc}\right]}^{⊤}T_{j}\left(s_{j}\right)\right)ds_{j}}\;. \end{array}$$

**Proof.**  See Appendix D.12. □

**Theorem** **5.** *Expectation Propagation: Given a TFFG*G=(V,E)*, consider the induced subgraph*G(b,j)*(Figure 5). Given the local polytope*

(65) $$\begin{array}{c} L\left(G\left(b,j\right)\right)=\left\{q_{b}\;s.t.\;(9a),\;and\;q_{j}\;s.t.\;(59)\;and\;(10)\right\}\;, \end{array}$$

*and*$\mu_{jb}\left(s_{j}\right)=\exp\;\left(\eta_{jb}^{⊤}T_{j}\left(s_{j}\right)\right)$*an exponential family message (from Lemma 6). Then, the local stationary solutions to* (15) *are given by*

(66a) $$\begin{array}{cc} q_{b}^{*}\left(s_{b}\right) & =\frac{f_{b}\left(s_{b}\right)\prod_{i\in E(b)}\mu_{ib}^{*}\left(s_{i}\right)}{\int f_{b}\left(s_{b}\right)\prod_{i\in E(b)}\mu_{ib}^{*}\left(s_{i}\right)ds_{b}} \end{array}$$

(66b) $$\begin{array}{cc} q_{j}^{*}\left(s_{j}\right) & =\frac{\exp\;\left({\left[\eta_{jb}^{*}+\eta_{jc}^{*}\right]}^{⊤}T_{j}\left(s_{j}\right)\right)}{\int \exp\;\left({\left[\eta_{jb}^{*}+\eta_{jc}^{*}\right]}^{⊤}T_{j}\left(s_{j}\right)\right)ds_{j}}\;, \end{array}$$

with $\eta_{jb}^{*},\eta_{jc}^{*}$ and $\mu_{jc}^{*}\left(s_{j}\right)$ being the fixed points of the iterations

$$\begin{array}{cc} {\tilde{\mu }}_{jc}^{\left(k\right)}\left(s_{j}\right) & =\int f_{b}\left(s_{b}\right)\prod_{\begin{array}{c} i\in E(b)\\ i\neq j \end{array}}\mu_{ib}^{\left(k\right)}\left(s_{i}\right)d\;s_{b\setminus j}\\ {\tilde{q}}_{j}^{\left(k\right)}\left(s_{j}\right) & =\frac{\mu_{jb}^{\left(k\right)}\left(s_{j}\right){\tilde{\mu }}_{jc}^{\left(k\right)}\left(s_{j}\right)}{\int \mu_{jb}^{\left(k\right)}\left(s_{j}\right){\tilde{\mu }}_{jc}^{\left(k\right)}\left(s_{j}\right)ds_{j}}\;. \end{array}$$

*By moment-matching on ${\tilde{q}}_{j}^{\left(k\right)}\left(s_{j}\right)$, we obtain the natural parameter ${\tilde{\eta }}_{j}^{\left(k\right)}$. The message update then follows from*

$$\begin{array}{cc} \eta_{jc}^{\left(k\right)} & ={\tilde{\eta }}_{j}^{\left(k\right)}-\eta_{jb}^{\left(k\right)}\\ \mu_{jc}^{\left(k+1\right)}\left(s_{j}\right) & \;=\;\exp\left(T_{j}{\left(s_{j}\right)}^{⊤}\eta_{jc}^{\left(k\right)}\right)\;. \end{array}$$

**Proof.**  See Appendix D.13. □

Moment-matching can be performed by solving [24] (Proposition 3.1)

$$\begin{array}{cc} \nabla_{\eta_{j}}\log Z_{j}\left(\eta_{j}\right) & =\int {\tilde{q}}_{j}\left(s_{j}\right)\;T_{j}\left(s_{j}\right)d\;s_{j} \end{array}$$

for $\eta_{j}$, where

$$\begin{array}{cc} Z_{j}\left(\eta_{j}\right) & =\int \exp\;\left(\eta_{j}^{⊤}T_{j}\left(s_{j}\right)\right)ds_{j}\;. \end{array}$$

In practice, for a Gaussian approximation, the natural parameters can be obtained by converting the matched mean and variance of ${\tilde{q}}_{j}\left(s_{j}\right)$ to the canonical form [18]. Computing the moments of ${\tilde{q}}_{j}\left(s_{j}\right)$ is often challenging due to lack of closed form solutions of the normalization constant. In order to address the computation of moments in EP, Ref. [44] proposes to evaluate challenging moments by quadrature methods. For multivariate random variables, moment-matching by spherical radial cubature would be advantageous as it will reduce the computational complexity [45]. Another popular way of evaluating the moments is through importance sampling [46] (Ch. 7) and [47].

Expectation propagation has been utilized in various applications ranging from time series estimation with Gaussian processes [48] to Bayesian learning with stochastic natural gradients [49]. When the likelihood functions for Gaussian process classification are not Gaussian, EP is often utilized [50] (Chapter 3). In [51], a message passing-based expectation propagation algorithm is developed for models that involve both continuous and discrete random variables. Perhaps the most practical applications of EP are in the context of probabilistic programming [52], where it is heavily used in real-world applications.

### 4.3. Hybrid Constraints

In this section, we consider hybrid methods that combine factorization and form constraints, and formalize some well-known algorithms in terms of message passing.

#### 4.3.1. Mean-Field Variational Laplace

Mean-field variational Laplace applies the mean-field factorization to the Laplace-approximated factor function. The appeal of this method is that all messages outbound from the Laplace-approximated factor can be represented by Gaussians.

**Theorem** **6.** *Mean-field variational Laplace: Given a TFFG*G=(V,E)*, consider the induced subgraph*G(b,j)*(Figure 13) with the Laplace-encoded factor*${\tilde{f}}_{b}$*as per* (53)*. We write the model* (1) *with substituted Laplace-encoded factor* ${\tilde{f}}_{b}$ *for* $f_{b}$*, as* $\tilde{f}$*. Furthermore, assume a naive mean-field factorization* l(b)={{i}foralli∈E(b)}*. Let* m∈l(b) *be the cluster where* j=m*. Given the local polytope of* (33)*, the local stationary solutions to*

(67) $$\begin{array}{cc} \left\{q_{b}^{m,*},q_{j}^{*}\right\} & =\arg\min_{L(G(b,j))}F\left[q,\tilde{f};{\hat{s}}_{b}\right]\;, \end{array}$$

*are given by*

$$\begin{array}{cc} q_{b}^{m,*}\left(s_{b}^{m}\right) & =q_{j}^{*}\left(s_{j}\right)=\frac{\mu_{jb}^{*}\left(s_{j}\right)\mu_{jc}^{*}\left(s_{j}\right)}{\int \mu_{jb}^{*}\left(s_{j}\right)\mu_{jc}^{*}\left(s_{j}\right)d\;s_{j}}\;, \end{array}$$

*where* $\mu_{jc}^{*}$ *represents the fixed points of the following iterations*

(68) $$\begin{array}{cc} \mu_{jc}^{\left(k+1\right)}\left(s_{j}\right) & =\exp\left(\int \left(\prod_{\begin{array}{c} i\in E(b)\\ i\neq j \end{array}}q_{i}^{\left(k\right)}\left(s_{i}\right)\right)\log{\tilde{f}}_{b}\left(s_{b};{\hat{s}}_{b}^{\left(k\right)}\right)d\;s_{b\setminus j}\right)\;, \end{array}$$

*with*

$$\begin{array}{c} {\hat{s}}_{b}^{\left(k\right)}=\arg\max_{s_{b}}\;\log q_{b}^{\left(k\right)}\left(s_{b}\right)\;. \end{array}$$

**Proof.**  See Appendix D.14. □

Conveniently, under these constraints, every outbound message from node *b* will be proportional to a Gaussian. Substituting the Laplace-approximated factor function, we obtain:

$$\begin{array}{cc} \log\mu_{jc}^{\left(k\right)}\left(s_{j}\right) & =\int \left(\prod_{\begin{array}{c} i\in E(b)\\ i\neq j \end{array}}q_{i}^{\left(k\right)}\left(s_{i}\right)\right)\;{\tilde{L}}_{b}\left(s_{b};{\hat{s}}_{b}^{\left(k\right)}\right)d\;s_{b\setminus j}+C\;. \end{array}$$

Resolving this expectation yields a quadratic form in $s_{j}$, which after completing the square leads to a proportionally Gaussian message $\mu_{jc}\left(s_{j}\right)$ . This argument holds for any edge adjacent to *b*, and therefore for all outbound messages from node *b*. Moreover, if the incoming messages are represented by Gaussians as well (e.g., because these are also computed under the mean-field variational Laplace constraint), then all beliefs on the adjacent edges to *b* will also be Gaussian. This significantly simplifies the procedure of computing the expectations, which illustrates the computational appeal of mean-field variational Laplace.

Mean-field variational Laplace is widely used in dynamic causal modeling [53] and more generally in cognitive neuroscience, partly because the resulting computations are deemed neurologically plausible [54,55,56].

#### 4.3.2. Expectation Maximization

Expectation Maximization (EM) can be viewed as a hybrid algorithm that combines a structured variational factorization with a Dirac-delta constraint, where the constrained value itself is optimized. Given a structured mean-field factorization l(a)⊆P(a), with a single-edge cluster m=j, then expectation maximization considers local factorizations of the form

(69) $$\begin{array}{c} q_{a}\left(s_{a}\right)=\delta \left(s_{j}-\theta_{j}\right)\;\prod_{\begin{array}{c} n\in l(a)\\ n\neq m \end{array}}q_{a}^{n}\left(s_{a}^{n}\right), \end{array}$$

where the belief for $s_{j}$ is constrained by a Dirac-delta distribution, similar to Section 4.2.1. In (69), however, the variable $s_{j}$ represents a random variable with (unknown) value $\theta_{j}\in R^{d}$, where *d* is the dimension of the random variable $s_{j}$. We explicitly use the notation $\theta_{j}$ (as opposed to ${\hat{s}}_{j}$ for the data constraint in Section 4.2.1) to clarify that this value is a parameter for the constrained belief over $s_{j}$ that will be optimized—that is, $\theta_{j}$ does not represent a model parameter in itself. To make this distinction even more explicit, in the context of optimization, we will refer to Dirac-delta constraints as point-mass constraints.

The factor-local free energy $F[q_{a},f_{a};\theta_{j}]$ then becomes a function of the $\theta_{j}$ parameter.

**Figure 14:**
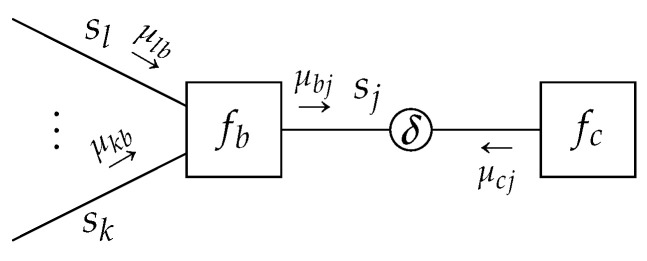
Visualization of a subgraph G(b,j) with indicated messages. The open circle indicates a point-mass constraint of the form $\delta (s_{j}-\theta_{j})$, where the value $\theta_{j}$ is optimized.

**Theorem** **7.** *Expectation maximization: Given a TFFG* G=(V,E) *, consider the induced subgraph* G(b,j) *(Figure 14) with a structured mean-field factorization* l(b)⊆P(b) *, with local clusters* n∈l(b) *. Let* m∈l(b) *be the cluster where* j=m *. Given the local polytope*

(70) $$\begin{array}{c} L\left(G\left(b,j\right)\right)=\left\{q_{b}^{n}\;for\;all\;n\in l\left(b\right)\;s.t.\;(29a)\right\}\;, \end{array}$$

*the local stationary solutions to*

$$\begin{array}{c} \theta_{j}^{*}=\arg\min_{L(G(b,j))}F\left[q,f;\theta_{j}\right]\;, \end{array}$$

*are given by the fixed points of*

(71a) $$\begin{array}{cc} \mu_{bj}^{\left(k+1\right)}\left(s_{j}\right) & =\exp\left(\int \left\{\prod_{\begin{array}{c} n\in l(b)\\ n\neq m \end{array}}q_{b}^{n,(k)}\left(s_{b}^{n}\right)\right\}\log f_{b}\left(s_{b}\right)d\;s_{b\setminus j}\right) \end{array}$$

(71b) $$\begin{array}{cc} \theta_{j}^{\left(k+1\right)} & =\arg\max_{s_{j}}\left(\log\mu_{bj}^{\left(k+1\right)}\left(s_{j}\right)+\log\mu_{cj}^{\left(k+1\right)}\left(s_{j}\right)\right)\;. \end{array}$$

**Proof.**  See Appendix D.15. □

Expectation maximization was formulated in [57] as an iterative method that optimizes log-expectations of likelihood functions, where each EM iteration is guaranteed to increase the expected log-likelihood. Moreover, under some differentiability conditions, the EM algorithm is guaranteed to converge [57] (Theorem 3). A detailed overview of EM for exponential families is available in [24] (Ch. 6). A formulation of EM in terms of message passing is given by [58], where message passing for EM is applied in a filtering and system identification context. In [58], derivations are based on [57] (Theorem 1), whereas our derivations directly follow from variational principles.

**Example** **4.** *Now suppose we do not know the angle*θ*for the state transition matrix*$A_{t}$*in Example 2 and would like to estimate the value of*θ*. Moreover, further suppose that we are interested in estimating the hyper-parameters for the prior*$m_{x_{0}}$*and*$V_{x_{0}}$*, as well as the precision matrix for the state transitions*$Q_{t}$*. For this purpose, we changed the constraints of* (25a) *into EM constraints in accordance with Theorem 7:*

(72a) $$\begin{array}{cc} q(x_{t-1},z_{t},A_{t}\left(\theta \right)) & =\delta \left(A_{t}\left(\theta \right)-A_{t}\left(\hat{\theta }\right)\right)q\left(z_{t}|x_{t-1},A_{t}\left(\theta \right)\right)q\left(x_{t-1}\right) \end{array}$$

(72b) $$\begin{array}{cc} q(x_{0},m_{x_{0}},V_{x_{0}}) & =q\left(x_{0}\right)\delta \left(m_{x_{0}}-{\hat{m}}_{x_{0}}\right)\delta \left(V_{x_{0}}-{\hat{V}}_{x_{0}}\right)\;, \end{array}$$

**Figure 15:**
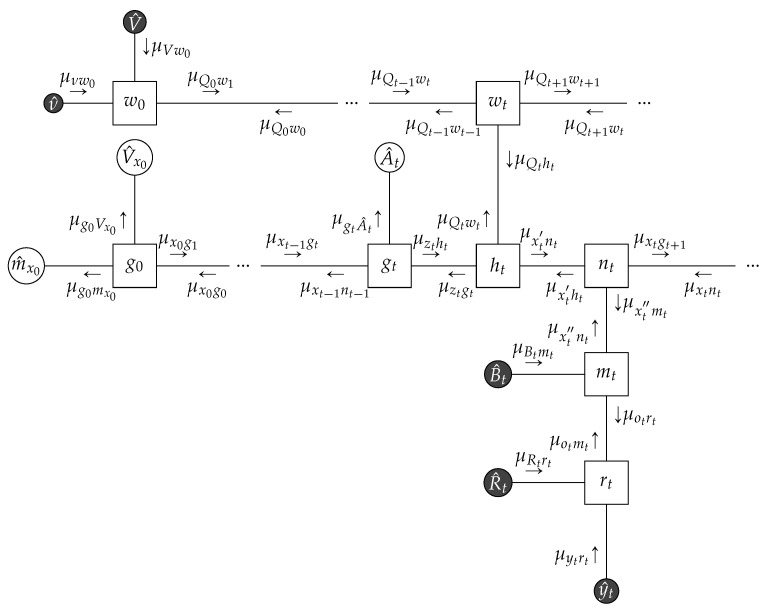
The FFG of the linear Gaussian state space model augmented with the EM constraints in Example 4.

**Figure 16:**
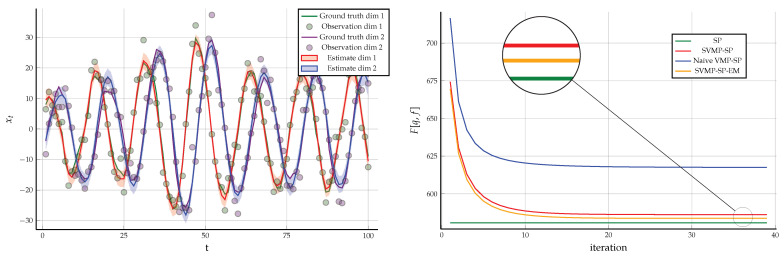
(**Left**) The small dots indicate the noisy observations that are synthetically generated by the linear state space model of (23) using matrices specified in (24). The posterior distribution of the hidden states inferred by structured variational message passing is depicted with shaded regions representing plus and minus one variances. The minimum of the evaluated Bethe free energy over iterations is F[q,f]=583.683. Moreover, the posterior distribution for the precision matrix is given by $Q∼W\left(\left[\begin{array}{cc} 0.00286 & 0.00038\\ 0.00038 & 0.0.00691 \end{array}\right],102.0\right)$. The EM estimates are θ=π/7.821, ${\hat{m}}_{x_{0}}=\left[7.23,-7.016\right]$ and ${\hat{V}}_{x_{0}}=\left[\begin{array}{cc} 11.028 & -1.926\\ -1.926 & 10.918 \end{array}\right]$. (**Right**) Free energy plots of the 4 algorithms discussed in Examples 1–4 on the same data set.

*where we optimize* $\hat{\theta },{\hat{V}}_{x_{0}}$ *and* ${\hat{m}}_{x_{0}}$ *with EM (*${\hat{V}}_{x_{0}}$ *is further constrained to be positive definite during the optimization procedure). With the addition of the new EM constraints, the resulting FFG is given in Figure 15. The hybrid message passing algorithm consists of structured variational messages around the factor* $h_{t}$*, and sum-product messages around* $w_{t}$*,* $n_{t}$*,* $m_{t}$ *and* $r_{t}$*, and EM messages around* $g_{0}$ *and* $g_{t}$*. We used identical observations as in the previous examples. The results for the hybrid SVMP-EM-SP algorithm are given in Figure 16 along with the evaluated Bethe free energy over all iterations.*

### 4.4. Overview of Message Passing Algorithms

In Section 4.1, Section 4.2 and Section 4.3, following a high-level recipe pioneered by [15], we presented first-principle derivations of some of the popular message passing-based inference algorithms by manipulating the local constraints of the Bethe free energy. The results are summarized in Table 1.

Crucially, the method of constrained BFE minimization goes beyond the reviewed algorithms. Through creating a new set of local constraints and following similar derivations based on variational calculus, one can obtain new message passing-based inference algorithms that better match the specifics of the generative model or application.

## 5. Scoring Models by Minimized Variational Free Energy

As discussed in Section 2.2, the variational free energy is an important measure of model performance. In Section 5.1 and Section 5.2, we discuss some problems that occur when evaluating the BFE on a TFFG. In Section 5.3, we propose an algorithm that evaluates the constrained BFE as a summation of local contributions on the TFFG.

### 5.1. Evaluation of the Entropy of Dirac-Delta Constrained Beliefs

For continuous variables, data and point-mass constraints, as discussed in Section 4.2.1 and Section 4.3.2 and Appendix A, collapse the information density to infinity, which leads to singularities in entropy evaluation [59]. More specifically, for a continuous variable $s_{j}$, the entropies for beliefs of the form $q_{j}\left(s_{j}\right)=\delta \left(s_{j}-{\hat{s}}_{j}\right)$ and $q_{a}\left(s_{a}\right)=q_{a|j}\left(s_{a\setminus j}|s_{j}\right)\delta \left(s_{j}-{\hat{s}}_{j}\right)$ both evaluate to −∞.

In variational inference, it is common to define the VFE only with respect to the latent (unobserved) variables [2] (Section 10.1). In contrast, in this paper, we explicitly define the BFE in terms of an iteration over all nodes and edges (7), which also includes non-latent beliefs in the BFE definition. Therefore, we define

$$\begin{array}{cc} q_{j}\left(s_{j}\right)=\delta \left(s_{j}-{\hat{s}}_{j}\right) & \Rightarrow H[q_{j}]≜0\;,\\ q_{a}\left(s_{a}\right)=q_{a|j}\left(s_{a\setminus j}|s_{j}\right)\delta \left(s_{j}-{\hat{s}}_{j}\right) & \Rightarrow H\left[q_{a}\right]≜H\left[q_{a\setminus j}\right]\;, \end{array}$$

where $q_{a|j}\left(s_{a\setminus j}|s_{j}\right)$ indicates the conditional belief and $q_{a\setminus j}\left(s_{a\setminus j}\right)$ is the joint belief. These definitions effectively remove the entropies for observed variables from the BFE evaluation. Note that although $q_{a\setminus j}\left(s_{a\setminus j}\right)$ is technically not a part of our belief set (7), it can be obtained by marginalization of $q_{a}\left(s_{a}\right)$ (9b).

### 5.2. Evaluation of Node-Local Free Energy for Deterministic Nodes

Another difficulty arises with the evaluation of the node-local free energy $F[q_{a}]$ for factors of the form

(73) $$\begin{array}{c} f_{a}\left(s_{a}\right)=\delta \left(h_{a}\left(s_{a}\right)\right)\;. \end{array}$$

This type of node function reflects deterministic operations, e.g., h(x,y,z)=z−x−y corresponds to the summation z=x+y. In this case, directly evaluating $F[q_{a}]$ again leads to singularities.

There are (at least) two strategies available in the literature that resolve this issue. The first strategy “softens” the Dirac-delta by re-defining:

$$\begin{array}{c} f_{a}\left(s_{a}\right)≜\frac{1}{\sqrt{2\pi \epsilon }}\exp\left(-\frac{1}{2\epsilon }h_{a}{\left(s_{a}\right)}^{2}\right)\;, \end{array}$$

with 0<ϵ≪1 [17]. A drawback of this approach is that it may alter the model definition in a numerically unstable way, leading to a different inference solution and variational free energy than originally intended.

The second strategy combines the deterministic factor $f_{a}$ with a neighboring stochastic factor $f_{b}$ into a new *composite* factor $f_{c}$, by marginalizing over a shared variable $s_{j}$, leading to [60]

$$\begin{array}{cc} f_{c}\left(s_{c}\right) & ≜\int \delta \left(h_{a}\left(s_{a}\right)\right)\;f_{b}\left(s_{b}\right)d\;s_{j}\;, \end{array}$$

where $s_{c}=\left\{s_{a}\cup s_{b}\right\}\setminus s_{j}$. This procedure has drawbacks for models that involve many deterministic factors—namely, the convenient model modularity and resulting distributed compatibility are lost when large groups of factors are compacted in model-specific composite factors. We propose here a third strategy.

**Theorem** **8.** *Let* $f_{a}\left(s_{a}\right)=\delta \left(h_{a}\left(s_{a}\right)\right)$ *, with* $h_{a}\left(s_{a}\right)=s_{j}-g_{a}\left(s_{a\setminus j}\right)$ *, and node-local belief* $q_{a}\left(s_{a}\right)=q_{j|a}\left(s_{j}|s_{a\setminus j}\right)\;q_{a\setminus j}\left(s_{a\setminus j}\right)$ *. Then, the node-local free energy evaluates to*

$$\begin{array}{cc} F[q_{a},f_{a}] & =\left\{\begin{array}{cc} -H[q_{a\setminus j}] & if\;q_{j|a}\left(s_{j}|s_{a\setminus j}\right)=\delta \left(s_{j}-g_{a}\left(s_{a\setminus j}\right)\right)\\ \infty & otherwise. \end{array}\right. \end{array}$$

**Proof.**  See Appendix D.16. □

An example that evaluates the node-local free energy for a non-trivial deterministic node can be found in Appendix C.

The equality node is a special case deterministic node, with a node function of the form (3). The argument of (Theorem 8) does not directly apply to this node. As the equality node function comprises two Dirac-delta functions, it can not be written in the form of Theorem 8. However, we can still reduce the node-local free energy contribution.

**Theorem** **9.** *Let* $f_{a}\left(s_{a}\right)=\delta \left(s_{j}-s_{i}\right)\;\delta \left(s_{j}-s_{k}\right)$ *, with node-local belief* $q_{a}\left(s_{a}\right)=q_{ik|j}\left(s_{i},s_{k}|s_{j}\right)\;q_{j}\left(s_{j}\right)$ *. Then, the node-local free energy evaluates to*

$$\begin{array}{cc} F[q_{a},f_{a}] & =\left\{\begin{array}{cc} -H[q_{j}] & if\;q_{ik|j}\left(s_{i},s_{k}|s_{j}\right)=\delta \left(s_{j}-s_{i}\right)\;\delta \left(s_{j}-s_{k}\right)\\ \infty & otherwise. \end{array}\right. \end{array}$$

**Proof.**  See Appendix D.17. □

### 5.3. Evaluating the Variational Free Energy

We propose here an algorithm that evaluates the BFE on a TFFG representation of a factorized model. The algorithm is based on the following results:

- The definitions for the computation of data-constrained entropies ensure that only variables with associated stochastic beliefs count towards the Bethe entropy. This makes the BFE evaluation consistent with Theorems 3 and 7, where the single-variable beliefs for observed variables are excluded from the BFE definition;
- We assume that a local mean-field factorization l(a) is available for each a∈V (Section 4.1). If the mean-field factorization is not explicitly defined, we assume l(a)={a} is the unfactored set;
- Deterministic nodes are accounted for by Theorem 8, which reduces the joint entropy to an entropy over the “inbound” edges. Although the belief over the “inbounds” $q_{a\setminus j}\left(s_{a\setminus j}\right)$ is not a term in the Bethe factorization (8), it can simply be obtained by marginalization of $q_{a}\left(s_{a}\right)$;
- The equality node is a special case, where we let the node entropy discount the degree of the associated variable in the original model definition. While the BFE definition on a TFFG (7) does not explicitly account for edge degrees, this mechanism implicitly corrects for “double-counting” [17]. In this case, edge selection for counting is arbitrary, because all associated edges are (by definition) constrained to share the same belief (Section 2.1, Theorem 9).

The decomposition of (7) shows that the BFE can be computed by an iteration over the nodes and edges of the graph. As some contributions to the BFE might cancel each other, the algorithm first tracks counting numbers $u_{a}$ for the average energies

$$\begin{array}{c} U_{a}\left[q_{a}\right]=-\int q_{a}\left(s_{a}\right)\log f_{a}\left(s_{a}\right)d\;s_{a}\;, \end{array}$$

and counting numbers $h_{k}$ for the (joint) entropies

$$\begin{array}{c} H\left[q_{k}\right]=-\int q_{k}\left(s_{k}\right)\log q_{k}\left(s_{k}\right)d\;s_{k}\;, \end{array}$$

which are ultimately combined and evaluated. We used an index *k* to indicate that the entropy computation may include not only the edges but a generic set of variables. We will give the definition of the set that *k* belongs to in Algorithm 1.
**Algorithm 1** Evaluation of the Bethe free energy on a Terminated Forney-style factor graph.
**given** a TFFG G=(V,E)**given** a local mean-field factorization l(a) for all a∈V**define**$q_{j}\left(s_{j}\right)=\delta \left(s_{j}-{\hat{s}}_{j}\right)\Rightarrow H\left[q_{j}\right]≜0$           ▹ Ignore entropy of Dirac-delta constrained beliefs**define**$q_{a}\left(s_{a}\right)=q_{a|j}\left(s_{a\setminus j}|s_{j}\right)\delta \left(s_{j}-{\hat{s}}_{j}\right)\Rightarrow H\left[q_{a}\right]≜H\left[q_{a\setminus j}\right]$ ▹ Reduce entropy of Dirac-delta constrained joint beliefs**define**K={a,a∖i,n,foralla∈V,i∈E(a),n∈l(a)} the set of (joint) belief indices**initialize** counting numbers $u_{a}=0$ for all a∈V, $h_{k}=0$ for all k∈K **for all** nodes a∈V **do**    **if** *a* is a stochastic node **then**        $u_{a}\;+\;\;=1$                        ▹ Count the average energy        **for all** clusters n∈l(a) **do**           $h_{n}\;+\;\;=1$                        ▹ Count the (joint) cluster entropy        **end for**    **else if** *a* is an equality node **then**        Select an edge j∈E(a)        $h_{j}\;+\;\;=1$                        ▹ Count the variable entropy    **else**                        ▹ Deterministic node *a*        Obtain the node function $f_{a}\left(s_{a}\right)=\delta \left(s_{j}-g_{a}\left(s_{a\setminus j}\right)\right)$        $h_{a\setminus j}\;+\;\;=1$                        ▹ Count the (joint) entropy of the inbounds    **end if****end for** **for all** edges i∈E **do**    $h_{i}\;-\;\;=1$                        ▹ Discount the variable entropy**end for** $U=\sum_{a\in V}u_{a}U_{a}\left[q_{a}\right]$$H=\sum_{k\in K}h_{k}H\left[q_{k}\right]$**return**F=U−H

## 6. Implementation of Algorithms and Simulations

We have developed a probabilistic programming toolbox *ForneyLab.jl* in the Julia language [61,62]. The majority of algorithms that are reviewed in Table 1 have been implemented in ForneyLab along with variety of demos (https://github.com/biaslab/ForneyLab.jl/tree/master/demo, accessed on 23 June 2021). ForneyLab is extendable and supports postulating new local constraints of the BFE for the creation of custom message passing-based inference algorithms.

In order to limit the length of this paper, we refer the reader to the demonstration folder of ForneyLab and to several of our previous papers with code. For instance, our previous work in [63] implemented a mean-field variational Laplace propagation for the hierarchical Gaussian filter (HGF) [64]. In the follow-up work [65], inference results improved by changing to structured factorization and moment-matching local constraints. In that case, modification of local constraints created a hybrid EP-VMP algorithm that better suited the model. Moreover, in [13], we formulated the idea of *chance constraints* in the form of violation probabilities leading to a new message passing algorithm that supports goal-directed behavior within the context of active inference. A similar line of reasoning led to improved inference procedures for auto-regressive models [66].

## 7. Related Work

Our work is inspired by the seminal work [17], which discusses the equivalence between the fixed points of the belief propagation algorithm [32] and the stationary points of the Bethe free energy. This equivalence is established through a Lagrangian formalism, which allows for the derivation of Generalized Belief Propagation (GBP) algorithms by introducing region-based graphs and the region-based (Kikuchi) free energy [16].

Region graph-based methods allows for overlapping clusters (Section 4.1) and thus offer a more generic message passing approach. The selection of appropriate regions (clusters), however, proves to be difficult, and the resulting algorithms may grow prohibitively complex. In this context, Ref. [67] addresses how to manipulate regions and manage the complexity of GBP algorithms. Furthermore, Ref. [68] also establishes a connection between GBP and expectation propagation (EP) by introducing structured region graphs.

The inspirational work of [15] derives message passing algorithms by minimization of α-divergences. The stationary points of α-divergences are obtained by a fixed point projection scheme. This projection scheme is reminiscent of the minimization scheme of the expectation propagation (EP) algorithm [18]. Compared to [15], our work focuses on a single divergence objective (namely, the VFE). The work of [12] derives the EP algorithm by manipulating the marginalization and factorization constraints of the Bethe free energy objective (see also Section 4.2.3). The EP algorithm is, however, not guaranteed to converge to a minimum of the associated divergence metric.

To address the convergence properties of the algorithms that are obtained by region graph methods, the outstanding work of [33] derives conditions on the region counting numbers that guarantee the convexity of the underlying objective. In general, however, the constrained Bethe free energy is not guaranteed to be convex and therefore the derived message passing updates are not guaranteed to converge.

## 8. Discussion

The key message in this paper is that a (variational) Bayesian model designer may tune the tractability-accuracy trade-off for evidence and posterior evaluation through constraint manipulation. It is interesting to note that the technique to derive message passing algorithms is always the same. We followed the recipe pioneered in [15] to derive a large variety of message passing algorithms solely through minimizing constrained Bethe free energy. This minimization leads to local fixed-point equations, which we can interpret as message passing updates on a (terminated) FFG. The presented lemmas showed how the constraints affect the Lagrangians locally. The presented theorems determined the stationary solutions of the Lagrangians and obtained the message passing equations. Thus, if a designer proposes a new set of constraints, then the first place to start is to analyze the effect on the Lagrangian. Once the effect of the constraint on the Lagrangian is known, then variational optimization may result in stationary solutions that can be obtained by a fixed-point iteration scheme.

In this paper, we selected the Forney-style factor graph framework to illustrate our ideas. FFGs are mathematically comparable to the more common bi-partite factor graphs that associate round nodes with variables and square nodes with factors [20]. Bi-partite factor graphs require two distinct types of message updates (one leaving variable nodes and one leaving factor nodes), while message passing on a (T)FFG requires only a single type of message update [69]. The (T)FFG paradigm thus substantially simplifies the derivations and resulting message passing update equations.

The message passing update rules in this paper are presented without guarantees on convergence of the (local) minimization process. In practice, however, algorithm convergence can be easily checked by evaluating the BFE (Algorithm 1) after each belief update.

In future work, we plan on extending the treatment of constraints to formulate sampling-based algorithms such as importance sampling and Hamiltonian Monte Carlo in a message passing framework. While introducing SVMP, we have limited the discussion to local clusters that are not overlapping. We plan to extend variational algorithms to include local clusters that are overlapping without altering the underlying free-energy objective or the graph structure [16,67].

## 9. Conclusions

In this paper, we formulated a message-passing approach to probabilistic inference by identifying local stationary solutions of a constrained Bethe free energy objective (Section 3 and Section 4). The proposed framework constructs a graph for the generative model and specifies local constraints for variational optimization in a local polytope. The constraints are then imposed on the variational objective by a Lagrangian construct. Unconstrained optimization of the Lagrangian then leads to local expressions of stationary points, which can be obtained by iterative execution of the resulting fixed point equations, which we identify with message passing updates.

Furthermore, we presented an approach to evaluate the BFE on a (terminated) Forney-style factor graph (Section 5). This procedure allows an algorithm designer to readily assess the performance of algorithms and models.

We have included detailed derivations of message passing updates (Appendix D) and hope that the presented formulation inspires the discovery of novel and customized message passing algorithms.

## Figures and Tables

**Table 1:** Relation between local constraints and derived message updates. The rows refer to different constraints that relate to factor–variable combinations, factors, and variables, respectively. Note that each message passing algorithm combines a set of constraints. Abbreviations: Sum-Product (SP), Structured Variational Message Passing (SVMP), Mean-Field Variational Message Passing (MFVMP), Data Constraint (DC), Laplace Propagation (LP), Mean-Field Variational Laplace (MFVLP), Expectation Maximization (EM), and Expectation Propagation (EP).

Local Constraint	SP	SVMP	MFVMP	DC	LP	MFVLP	EM	EP
Normalization	✓	✓	✓	✓	✓	✓	✓	✓
Marginalization	✓	✓	✓	✓	✓	✓	✓	✓
Moment-Matching								✓
Structured Mean-Field		✓					✓	
Naive Mean-Field			✓			✓		
Laplace Approximation					✓	✓		
Dirac-delta				✓			✓	
Estimation							✓	

**Figure A1:**
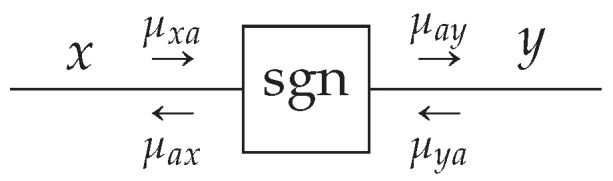
Messages around a “sign” node.

## Abbreviations

The following abbreviations are used in this manuscript:

BFE	Bethe Free Energy
BP	Belief Propagation
DC	Data Constraint
EM	Expectation Maximization
EP	Expectation Propagation
FFG	Forney-style Factor Graph
GBP	Generalized Belief Propagation
LP	Laplace Propagation
MFVLP	Mean-Field Variational Laplace
MFVMP	Mean-Field Variational Message Passing
NLE	Negative Log-Evidence
TFFG	Terminated Forney-style Factor Graph
VFE	Variational Free Energy
VMP	Variational Message Passing
SVMP	Structured Variational Message Passing
SP	Sum-Product

## Appendix A. Free Energy Minimization by Variational Inference

In this section, we present a pedagogical example of inductive inference. After we establish an intuition, we apply the same principles to a more general context in the further sections. We follow Caticha [42,70], who showed that a constrained free energy functional can be interpreted as a principled objective measure for inductive reasoning, see also [71,72]. The calculus of variations offers a principled method for optimizing this free energy functional.

In this section, we assume an example model

(A1) $$\begin{array}{c} f\left(y,\theta \right)=f_{y}\left(y,\theta \right)\;f_{\theta }\left(\theta \right)\;, \end{array}$$

with observed variables y and a single parameter θ.

We define the (variational) free energy (VFE) as

(A2) $$\begin{array}{cc} F[q,f] & =\;\int \int \;q\left(y,\theta \right)\log\frac{q(y,\theta )}{f(y,\theta )}d\;yd\;\theta \;. \end{array}$$

The goal is to find a posterior

(A3) $$\begin{array}{c} q^{*}=\arg\min_{q\in Q}F\left[q,f\right]\; \end{array}$$

that minimizes the free energy subject to some pre-specified constraints. These constraints may include form or factorization constraints on *q* (to be discussed later) or relate to observations of a signal y.

As an example, assume that we obtained some measurements $y=\hat{y}$ and wish to obtain a posterior marginal belief $q^{*}\left(\theta \right)$ over the parameter. We can then incorporate the data in the form of a data constraint

(A4) $$\begin{array}{c} \int q\left(y,\theta \right)d\;\theta =\delta \left(y-\hat{y}\right)\;, \end{array}$$

where δ defines a Dirac-delta. The *constrained* free energy can be rewritten by including Lagrange multipliers as

(A5) $$\begin{array}{c} L\left[q,f\right]=F\left[q,f\right]+\gamma \left(\;\int \int \;q(y,\theta )d\;yd\;\theta -1\right)+\int \lambda \left(y\right)\left(\int q\left(y,\theta \right)d\;\theta -\delta \left(y-\hat{y}\right)\right)d\;y\;, \end{array}$$

where the first term specifies the (to be minimized) free energy objective, the second term a normalization constraint, and the third term the data constraint. Optimization of (A5) can be performed using variational calculus.

Variational calculus considers the impact of a variation in q(y,θ) on the Lagrangian L[q,f]. We define the variation as

$$\begin{array}{c} \delta q\left(y,\theta \right)\overset{\Delta }{=}\epsilon \phi \left(y,\theta \right)\;, \end{array}$$

where ϵ→0, and ϕ is a continuous and differentiable “test” function. The fundamental theorem of variational calculus states that the stationary solutions $q^{*}$ are obtained by setting δL/δq=0, where the functional derivative δL/δq is implicitly defined by Appendix D in [2]:

(A6) $$\begin{array}{cc} \frac{d\;L[q+\epsilon \phi ,f]}{d\;\epsilon }{|}_{\epsilon =0} & =\;\int \int \;\frac{\delta L}{\delta q}\left(y,\theta \right)\;\phi \left(y,\theta \right)d\;yd\;\theta \;. \end{array}$$

Equation (A6) provides a way to derive the functional derivative through ordinary differentiation. For example, we take the Lagrangian defined by (A5) and work out the left hand side of (A6):

(A7a) $$\begin{array}{cc} \frac{d\;L[q+\epsilon \phi ,f]}{d\;\epsilon }{|}_{\epsilon =0} & =\frac{d\;F[q+\epsilon \phi ,f]}{d\;\epsilon }{|}_{\epsilon =0}+\frac{d}{d\;\epsilon }\gamma \;\int \int \;\left(q+\epsilon \phi \right)d\;yd\;\theta {|}_{\epsilon =0}+\frac{d}{d\;\epsilon }\int \lambda \left(y\right)\int \left(q+\epsilon \phi \right)d\;\theta d\;y{|}_{\epsilon =0}\\ \; & =\;\int \int \;\frac{d}{d\;\epsilon }\left(\left(q+\epsilon \phi \right)\log\frac{\left(q+\epsilon \phi \right)}{f}\right){|}_{\epsilon =0}d\;yd\;\theta +\gamma \;\int \int \;\frac{d}{d\;\epsilon }\left(q+\epsilon \phi \right){|}_{\epsilon =0}d\;yd\;\theta \end{array}$$

(A7b) $$\begin{array}{cc} \; & \;+\int \lambda \left(y\right)\int \frac{d}{d\;\epsilon }\left(q+\epsilon \phi \right){|}_{\epsilon =0}d\;\theta d\;y \end{array}$$

(A7c) $$\begin{array}{cc} \; & =\;\int \int \;[\underset{\delta L[q,f]/\delta q}{\underset{︸}{\log\frac{q(y,\theta )}{f(y,\theta )}+1+\gamma +\lambda \left(y\right)}}]\phi \left(y,\theta \right)d\;yd\;\theta \;. \end{array}$$

Note that, since (A7c) has been written in similar form as (A6), it is easy to identify the functional derivative. This procedure is one of many ways to obtain the functional derivatives [73].

Setting δL[q,f]/δq=0 we find the stationary solution as

(A8a) $$\begin{array}{cc} q^{*}\left(y,\theta \right) & =\exp(-1-\gamma -\lambda (y))\;f(y,\theta ) \end{array}$$

(A8b) $$\begin{array}{cc} \; & =\frac{1}{Z}\exp\left(-\lambda \left(y\right)\right)f\left(y,\theta \right)\;, \end{array}$$

with Z=∫∫exp(−λ(y))f(y,θ)dydθ=exp(γ+1). In order to determine the Lagrange multipliers γ and λ(y), we must substitute the stationary solution (A8b) back into the constraints. The normalization constraint evaluates to

(A9) $$\begin{array}{c} \frac{1}{Z}\;\int \int \;\exp\left(-\lambda \left(y\right)\right)f\left(y,\theta \right)d\;yd\;\theta =1. \end{array}$$

We find that (A9) is always satisfied since Z=∫∫exp(−λ(y))f(y,θ)dydθ by definition. Note, however, that the computation of the normalization constant still depends on the undetermined Lagrange multiplier λ(y).

The data constraint evaluates to

(A10) $$\int q^{*}\left(y,\theta \right)d\;\theta =\frac{1}{Z}\exp\left(-\lambda \left(y\right)\right)\int f\left(y,\theta \right)d\;\theta =\delta \left(y-\hat{y}\right)$$

which can be rewritten as

(A11) $$\frac{\exp(-\lambda (y))}{Z}=\frac{\delta (y-\hat{y})}{\int f(y,\theta )d\;\theta }\;.$$

Equation (A11) shows that λ(y) can satisfy this constraint only if it is proportional to $\delta (y-\hat{y})$. Indeed, substitution of (A11) into (A8b) gives

$$\begin{array}{c} q^{*}\left(y,\theta \right)=\frac{f(y,\theta )}{\int f(y,\theta )d\;\theta }\delta \left(y-\hat{y}\right)\;, \end{array}$$

and the posterior for the parameters evaluates to

$$\begin{array}{cc} q^{*}\left(\theta \right) & =\int q^{*}\left(y,\theta \right)dy\\ \; & =\int \frac{f(y,\theta )}{\int f(y,\theta )d\;\theta }\delta \left(y-\hat{y}\right)dy\\ \; & =\frac{f(\hat{y},\theta )}{\int f(\hat{y},\theta )d\;\theta }\\ \; & =\frac{f_{y}\left(\hat{y},\theta \right)f_{\theta }\left(\theta \right)}{\int f_{y}\left(\hat{y},\theta \right)f_{\theta }\left(\theta \right)d\;\theta }\;, \end{array}$$

which we recognize as the Bayes rule.

Note that the Bayes rule was derived here as a special case of constrained variational free energy minimization when data constraints are present. This derivation of the Bayes rule seems unnecessarily tedious but the value of this approach to inductive inference is that the same principle applies when other (not data) constraints on *q* are present.

## Appendix B. Lagrangian Optimization and the Dual Problem

With the addition of Lagrange multipliers to the Bethe functional, the resulting Lagrangian depends both on the variational distribution q(s) and the Lagrange multipliers Ψ(s). Formally, the introduction of the Lagrange multipliers allows us to rewrite the constrained optimization on the local polytope as an unconstrained optimization. We follow [33], and write

$$\begin{array}{c} \min_{q\in L(G)}F\left[q\right]=\min_{q}\max_{\Psi }L\left[q,\Psi \right]\;. \end{array}$$

Weak duality [74] (Chapter 5) then states that

$$\begin{array}{c} \min_{q}\max_{\Psi }L\left[q,\Psi \right]\geq \max_{\Psi }\min_{q}L\left[q,\Psi \right]\;. \end{array}$$

The minimization with respect to *q* then yields a solution that depends on the Lagrange multipliers, as

$$\begin{array}{c} q^{*}\left(s;\Psi \right)=\arg\min_{q}L\left[q,\Psi \right]\;. \end{array}$$

For any given *q* the Lagrangian is concave in Ψ. Therefore, substituting $q^{*}$ in the Lagrangian, the maximization over $L[q^{*},\Psi ]$ yields the unique solution

$$\begin{array}{c} \Psi^{*}\left(s\right)=\arg\max_{\Psi }L\left[q^{*},\Psi \right]\;. \end{array}$$

Stationary solutions are then given by

$$\begin{array}{c} q^{*}\left(s;\Psi^{*}\right)=\arg\min_{q\in L(G)}F\left[q\right]\;. \end{array}$$

In the current paper, we consider factorized *q*’s (e.g., (8)), and consider variations with respect to the individual factors. We then need to show that the combined stationary points of the individual factors also constitute a stationary point of the total objective.

Consider a Lagrangian having multiple arguments, i.e.,

(A12) $$\begin{array}{cc} L[q] & =L[q_{1},\ldots ,q_{n},\ldots ,q_{N}] \end{array}$$

(A13) $$\begin{array}{cc} q & ≜{\left[q_{1},\ldots ,q_{N}\right]}^{⊤}\;. \end{array}$$

We want to determine the first total variation of the Lagrangian given by

(A14) $$\begin{array}{cc} \delta L & =L[q+\epsilon \phi ]-L[q] \end{array}$$

(A15) $$\begin{array}{cc} \phi (s) & ≜{\left[\phi_{1}\left(s\right),\cdots ,\phi_{N}\left(s\right)\right]}^{⊤}\;. \end{array}$$

By a Taylor series expansion on ϵ we obtain [73] (A.14) and [75] (Equation (23.2))

(A16) $$L\left[q+\epsilon \phi \right]-L\left[q\right]=\sum_{k=1}^{K}\frac{1}{k!}\frac{d}{d\epsilon^{k}}\left(L^{k}\left[q+\epsilon \phi \right]\right)\epsilon^{k}+O\left(\epsilon^{K+1}\right)\;.$$

Omitting all terms higher than the first order, we obtain the first variation as

(A17) $$\delta L=\frac{d}{d\epsilon }\left(L[q+\epsilon \phi ]\right)\epsilon \;.$$

Rearranging the terms and letting ϵ vanish, we obtain the following expression

(A18) $$\lim_{\epsilon \to 0}\frac{\delta L}{\epsilon }=\frac{d}{d\epsilon }\left(L[q+\epsilon \phi ]\right){|}_{\epsilon =0}\;.$$

Let us assume that the Frechet derivative exists [73] such that we can obtain the following integral representation (It should be noted that this integral expression is not always possible for a generic Lagrangian. That is why we need to assume that the Frechet derivative exists)

(A19) $$\frac{d}{d\epsilon }\left(L[q+\epsilon \phi ]\right){|}_{\epsilon =0}=\int \phi {\left(s\right)}^{⊤}\frac{\delta L}{\delta q}ds$$

where $\frac{\delta L}{\delta q}$ is the variational derivative

(A20) $$\begin{array}{cc} \frac{\delta L}{\delta q} & ={\left[\frac{\delta L}{\delta q_{1}},\ldots ,\frac{\delta L}{\delta q_{N}}\right]}^{⊤} \end{array}$$

(A21) $$\begin{array}{cc} \delta q_{n} & =\epsilon \phi_{n}\left(s\right)\;. \end{array}$$

This means that (A19) can be written as [75] (Equation (22.5)) (Here, we use a more generic Lagrangian and our notation is different than in [75]; howeverm the expression is motivated again by a Taylor series expansion on ϵ)

(A22) $$\lim_{\epsilon \to 0}\frac{\delta L}{\epsilon }=\frac{d}{d\epsilon }\left(L[q+\epsilon \phi ]\right){|}_{\epsilon =0}=\sum_{n}\int \phi \left(s\right)\frac{\delta L}{\delta q_{n}}ds\;.$$

Fundamental theorem of variational calculus states that in order for a point to be stationary, the first variation needs to vanish. In order for the first variation to vanish, it is sufficient to have vanishing of the variational derivatives

(A23) $$\frac{\delta L}{\delta q_{n}}=0\;\;\mathrm{for}\;\mathrm{every}\;\;n=1,\ldots ,N\;.$$

Vanishing of individual variational derivatives will mean that that the local stationary points will also correspond to a global stationary point.

## Appendix C. Local Free Energy Example for a Deterministic Node

Theorem 8 tells us how to evaluate the node-local free energy for a deterministic node. As an example, consider the node function $f_{a}\left(y,x\right)=\delta \left(y-\mathrm{sgn}\left(x\right)\right)$, with y∈{−1,1} and x∈R as depicted in Figure A1.

Interestingly, there is information loss in this node because the “sign” mapping is not bijective. Given an incoming Bernoulli distributed message $\mu_{ya}\left(y\right)=Ber\left(y|p\right)$, the backward outgoing message is derived as

$$\begin{array}{cc} \mu_{ax}\left(x\right) & =\int \mu_{ya}\left(y\right)\;\delta \left(y-\mathrm{sgn}\left(x\right)\right)d\;y\\ \; & =\left\{\begin{array}{cc} p & \;\mathrm{if}\;x\geq 0\\ 1-p & \;\mathrm{if}\;x<0\;. \end{array}\right. \end{array}$$

Given a Gaussian distributed incoming message $\mu_{xa}\left(x\right)=N\;\left(x|m,\vartheta \right)$, the resulting belief then becomes

$$\begin{array}{cc} q_{x}\left(x\right) & =\frac{\mu_{xa}\left(x\right)\;\mu_{ax}\left(x\right)}{\int \mu_{xa}\left(x\right)\;\mu_{ax}\left(x\right)d\;x}\\ \; & =\left\{\begin{array}{cc} \frac{p}{p+\Phi -2p\Phi }\;N\;\left(x|m,\vartheta \right) & \;\mathrm{if}\;x\geq 0\\ \frac{1-p}{p+\Phi -2p\Phi }\;N\;\left(x|m,\vartheta \right) & \;\mathrm{if}\;x<0\;, \end{array}\right. \end{array}$$

with $\Phi =\int_{-\infty }^{0}N\;\left(x|m,\vartheta \right)d\;x$. We define a truncated Gaussian distribution as

$$\begin{array}{c} T\;\left(x|m,\vartheta ,a,b\right)=\left\{\begin{array}{cc} \frac{1}{\Phi (a,b;m,\vartheta )}N\;\left(x|m,\vartheta \right) & \;\mathrm{if}\;a\leq x\leq b\;,\\ 0 & \;\mathrm{otherwise}, \end{array}\right. \end{array}$$

with $\Phi \left(a,b;m,\vartheta \right)=\int_{a}^{b}N\;\left(x|m,\vartheta \right)d\;x$. This leads to

$$\begin{array}{cc} q_{x}\left(x\right) & =\underset{K}{\underset{︸}{\frac{p(1-\Phi )}{p+\Phi -2p\Phi }}}T\;\left(x|m,\vartheta ,-\infty ,0\right)+\underset{1-K}{\underset{︸}{\frac{(1-p)\Phi }{p+\Phi -2p\Phi }}}T\;\left(x|m,\vartheta ,0,\infty \right)\;, \end{array}$$

as a truncated Gaussian mixture.

The node-local free energy then evaluates to

$$\begin{array}{cc} F[q_{a},f_{a}] & =-H[q_{x}]\\ \; & =\int_{-\infty }^{0}q_{x}\left(x\right)\log q_{x}\left(x\right)d\;x+\int_{0}^{\infty }q_{x}\left(x\right)\log q_{x}\left(x\right)d\;x\\ \; & =-KH[T\;(m,\vartheta ,-\infty ,0)]+K\log K-(1-K)H[T\;(m,\vartheta ,0,\infty )]+(1-K)\log(1-K)\\ \; & =-KH[T\;(m,\vartheta ,-\infty ,0)]-(1-K)H[T\;(m,\vartheta ,0,\infty )]-H[Ber(K)]\;, \end{array}$$

as a weighted sum of entropies, which can be computed analytically.

## Appendix D. Proofs

### Appendix D.1. Proof of Lemma 1

**Proof.** We apply the variation $\epsilon \phi_{b}$ to $q_{b}$ and, as discussed in Appendix A, we can identify the functional derivative $\delta L_{b}/\delta q_{b}$ through ordinary differentiation as

$$\begin{array}{cc} \frac{dL_{b}\left[q_{b}+\epsilon \phi_{b},f_{b}\right]}{d\epsilon }{|}_{\epsilon =0} & =\int (\overset{\delta L_{b}/\delta q_{b}}{\overset{︷}{\log\frac{q_{b}\left(s_{b}\right)}{f_{b}\left(s_{b}\right)}+1+\psi_{b}-\sum_{i\in E(b)}\lambda_{ib}\left(s_{i}\right)}})\phi_{b}\left(s_{b}\right)d\;s_{b}\;. \end{array}$$

Setting the functional derivative to zero and identifying

(A24) $$\begin{array}{cc} \mu_{ib}\left(s_{i}\right) & =\exp\left(\lambda_{ib}\left(s_{i}\right)\right) \end{array}$$

(A25) $$\begin{array}{cc} \psi_{b} & =\log\int f_{b}\left(s_{b}\right)\prod_{i\in E(b)}\mu_{ib}\left(s_{i}\right)ds_{b}-1 \end{array}$$

yields the stationary solutions (18) in terms of Lagrange multipliers that are to be determined. □

### Appendix D.2. Proof of Lemma 2

**Proof.** We follow the same procedure as in Appendix D.1, where we apply a variation $\epsilon \phi_{j}$ to $q_{j}$ (instead of $q_{b}$), and identify the functional derivative $\delta L_{j}/\delta q_{j}$ through

$$\begin{array}{cc} \frac{dL_{j}\left[q_{j}+\epsilon \phi_{j}\right]}{d\epsilon }{|}_{\epsilon =0} & =\int (\overset{\delta L_{j}/\delta q_{j}}{\overset{︷}{-\log q_{j}\left(s_{j}\right)-1+\psi_{j}+\sum_{a\in V(j)}\lambda_{ja}\left(s_{j}\right)}})\phi_{j}\left(s_{j}\right)d\;s_{j}\;. \end{array}$$

As the TFFG is terminated, each edge has 2 degrees and the node-induced edge set has only 2 factors, which we denote by $f_{b}$ and $f_{c}$. Then, setting the functional derivative to zero and identifying

(A26) $$\begin{array}{cc} \mu_{ja}\left(s_{j}\right) & =\exp\left(\lambda_{ja}\left(s_{j}\right)\right) \end{array}$$

(A27) $$\begin{array}{cc} \psi_{j} & =-\log\int \mu_{jb}\left(s_{j}\right)\mu_{jc}\left(s_{j}\right)ds_{j}+1 \end{array}$$

yields the stationary solution of (20) in terms of the Lagrange multipliers. □

### Appendix D.3. Proof of Theorem 1

**Proof.** The local polytope of (14) constructs the Lagrangians of (17) and (19). Substituting the stationary solutions from Lemmas 1 and 2 in the marginalization constraint,

$$\begin{array}{cc} q_{j}\left(s_{j}\right) & =\int q_{b}\left(s_{b}\right)d\;s_{b\setminus j}\;, \end{array}$$

we obtain the following relation

$$\begin{array}{cc} \frac{\mu_{jb}\left(s_{j}\right)\mu_{jc}\left(s_{j}\right)}{Z_{j}} & =\frac{1}{Z_{b}}\int f_{b}\left(s_{b}\right)\prod_{i\in E(b)}\mu_{ib}\left(s_{i}\right)d\;s_{b\setminus j}\;, \end{array}$$

where we defined the following normalization constants to ensure that the computed marginals are normalized:

$$\begin{array}{cc} Z_{j} & =\int \mu_{jb}\left(s_{j}\right)\mu_{jc}\left(s_{j}\right)ds_{j}\\ Z_{b} & =\int f_{b}\left(s_{b}\right)\prod_{i\in E(b)}\mu_{ib}\left(s_{i}\right)ds_{b}\;. \end{array}$$

Extracting $\mu_{jb}$ from the integral

(A28) $$\begin{array}{cc} \frac{\mu_{jb}\left(s_{j}\right)\mu_{jc}\left(s_{j}\right)}{Z_{j}} & =\frac{\mu_{jb}\left(s_{j}\right)}{Z_{b}}\int f_{b}\left(s_{b}\right)\prod_{\begin{array}{c} i\in E(b)\\ i\neq j \end{array}}\mu_{ib}\left(s_{i}\right)d\;s_{b\setminus j}\;,\\ \mu_{jc}\left(s_{j}\right) & =\frac{Z_{j}}{Z_{b}}\int f_{b}\left(s_{b}\right)\prod_{\begin{array}{c} i\in E(b)\\ i\neq j \end{array}}\mu_{ib}\left(s_{i}\right)d\;s_{b\setminus j} \end{array}$$

and cancelling $\mu_{jb}$ on both sides then yields the condition on the functional form of the message $\mu_{jc}$. We now need to show that the fixed points of (22) satisfy (A28). Let us assume that the fixed points exist, such that $\mu_{jc}^{\left(k\right)}=\mu_{jc}^{\left(k+1\right)}$ for some *k*. Then, we want to show that at the fixed points the following equality holds:

$$\mu_{jc}^{\left(k\right)}\left(s_{j}\right)=\frac{Z_{j}^{\left(k\right)}}{Z_{b}^{\left(k\right)}}\int f_{b}\left(s_{b}\right)\prod_{\begin{array}{c} i\in E(b)\\ i\neq j \end{array}}\mu_{ib}^{\left(k\right)}\left(s_{i}\right)d\;s_{b\setminus j}\;.$$

Substituting (22), we need to show that

$$\mu_{jc}^{\left(k\right)}\left(s_{j}\right)=\frac{Z_{j}^{\left(k\right)}}{Z_{b}^{\left(k\right)}}\mu_{jc}^{\left(k+1\right)}\left(s_{j}\right)\;.$$

Since $\mu_{jc}^{\left(k\right)}=\mu_{jc}^{\left(k+1\right)}$, we can rearrange

$$\mu_{jc}^{\left(k\right)}\left(1-\frac{Z_{j}^{\left(k\right)}}{Z_{b}^{\left(k\right)}}\right)=0\;.$$

From $Z_{b}$, we obtain

$$\begin{array}{cc} Z_{b}^{\left(k\right)} & =\int \mu_{jb}^{\left(k\right)}\left(s_{j}\right)\int f_{b}\left(s_{b}\right)\prod_{\begin{array}{c} i\in E(b)\\ i\neq j \end{array}}\mu_{ib}^{\left(k\right)}\left(s_{i}\right)d\;s_{b\setminus j}ds_{j}\\ \; & =\int \mu_{jb}^{\left(k\right)}\left(s_{j}\right)\mu_{jc}^{\left(k+1\right)}\left(s_{j}\right)ds_{j}\\ \; & =\int \mu_{jb}^{\left(k\right)}\left(s_{j}\right)\mu_{jc}^{\left(k\right)}\left(s_{j}\right)ds_{j}\\ \; & =Z_{j}^{\left(k\right)}\;, \end{array}$$

which implies that the fixed points satisfy the desired condition. This proves that the stationary solutions to the BFE within the local polytope can be obtained as fixed points of the sum-product update equations. □

### Appendix D.4. Proof of Lemma 3

**Proof.** Substituting the definition of (32), we can re-write the second term of Lagrangian (30) as

$$\begin{array}{cc} \int \left\{\prod_{n\in l(b)}q_{b}^{m}\left(s_{b}^{m}\right)\right\}\log f_{b}\left(s_{b}\right)d\;s_{b}\; & =\int q_{b}^{m}\left(s_{b}^{m}\right)\left(\int \left\{\prod_{\begin{array}{c} n\in l(b)\\ n\neq m \end{array}}q_{b}^{n}\left(s_{b}^{n}\right)\right\}\log f_{b}\left(s_{b}\right)d\;s_{b}^{\setminus m}\right)d\;s_{b}^{m}\\ \; & =\int q_{b}^{m}\left(s_{b}^{m}\right)\log{\tilde{f}}_{b}^{m}\left(s_{b}^{m}\right)d\;s_{b}^{m}\;. \end{array}$$

We apply the variation $\epsilon \phi_{b}^{m}$ to $q_{b}^{m}$ and identify the functional derivative $\delta L_{b}^{m}/\delta q_{b}^{m}$, as

$$\begin{array}{cc} \frac{dL_{b}^{m}\left[q_{b}^{m}+\epsilon \phi_{b}^{m}\right]}{d\epsilon }{|}_{\epsilon =0} & =\int \left(\overset{\delta L_{b}^{m}/\delta q_{b}^{m}}{\overset{︷}{\log\frac{q_{b}^{m}\left(s_{b}^{m}\right)}{{\tilde{f}}_{b}^{m}\left(s_{b}^{m}\right)}+1+\psi_{b}^{m}-\sum_{i\in m}\lambda_{ib}\left(s_{i}\right)}}\right)\phi_{b}^{m}\left(s_{b}^{m}\right)d\;s_{b}^{m}\;, \end{array}$$

whose functional form we recognize from Appendix D.1. Setting the functional derivative to zero and again identifying $\mu_{ib}\left(s_{i}\right)=\exp\lambda_{ib}\left(s_{i}\right)$, yields the stationary solutions of (31). □

### Appendix D.5. Proof of Theorem 2

**Proof.** The local polytope of (33) constructs the Lagrangians $L_{b}^{m}$ and $L_{j}$ as (30) and (19), respectively. We substitute the stationary solutions of Lemmas 2 and 3 in the local marginalization constraint (29b), which yields

$$\begin{array}{c} q_{j}\left(s_{j}\right)=\int q_{b}^{m}\left(s_{b}^{m}\right)d\;s_{b\setminus j}^{m}\;. \end{array}$$

Following the structure of the proof in Appendix D.3, we obtain the following condition for the stationary solutions in terms of messages:

(A29) $$\begin{array}{cc} \frac{\mu_{jb}\left(s_{j}\right)\mu_{jc}\left(s_{j}\right)}{Z_{j}} & \;=\;\frac{\mu_{jb}\left(s_{j}\right)}{Z_{b}^{m}}\int {\tilde{f}}_{b}^{m}\left(s_{b}^{m}\right)\prod_{\begin{array}{c} i\in m\\ i\neq j \end{array}}\mu_{ib}\left(s_{i}\right)ds_{b\setminus j}^{m}\;\\ \frac{\mu_{jc}\left(s_{j}\right)}{Z_{j}} & \;=\;\frac{1}{Z_{b}^{m}}\int {\tilde{f}}_{b}^{m}\left(s_{b}^{m}\right)\prod_{\begin{array}{c} i\in m\\ i\neq j \end{array}}\mu_{ib}\left(s_{i}\right)ds_{b\setminus j}^{m}\;. \end{array}$$

Now we want to show that the fixed points of the message updates (36) satisfy (A29). Let us assume that the fixed points exists for some *k* such that $\mu_{jc}^{\left(k+1\right)}=\mu_{jc}^{\left(k\right)}$. Then, we will show that the fixed points satisfy

(A30) $$\frac{\mu_{jc}^{\left(k\right)}\left(s_{j}\right)}{Z_{j}^{\left(k\right)}}=\frac{1}{Z_{b}^{m,(k)}}\int {{\tilde{f}}_{b}}^{m,(k)}\left(s_{b}^{m}\right)\prod_{\begin{array}{c} i\in m\\ i\neq j \end{array}}\mu_{ib}^{\left(k\right)}\left(s_{i}\right)ds_{b\setminus j}^{m}\;\;.$$

Similar to Appendix D.3, it will suffice to show that $Z_{b}^{m,(k)}=Z_{j}^{\left(k\right)}$ at the fixed points. Arranging the order of integration in normalization constant $Z_{b}^{m,(k)}$, we obtain

$$\begin{array}{cc} Z_{b}^{m,(k)} & =\int \mu_{jb}^{\left(k\right)}\left(s_{j}\right)\int {{\tilde{f}}_{b}}^{m,(k)}\left(s_{b}^{m}\right)\prod_{\begin{array}{c} i\in m\\ i\neq j \end{array}}\mu_{ib}^{\left(k\right)}\left(s_{i}\right)ds_{b\setminus j}^{m}ds_{j}\\ \; & =\int \mu_{jb}^{\left(k\right)}\left(s_{j}\right)\mu_{jc}^{\left(k+1\right)}\left(s_{j}\right)ds_{j}\\ \; & =\int \mu_{jb}^{\left(k\right)}\left(s_{j}\right)\mu_{jc}^{\left(k\right)}\left(s_{j}\right)ds_{j}\\ \; & =Z_{j}^{\left(k\right)}\;. \end{array}$$

By the same line of reasoning as in Appendix D.3, this shows that the fixed points of the message updates (36) leads to stationary distributions of the Bethe free energy with structured factorization constraints. □

### Appendix D.6. Proof of Corollary 1

**Proof.** For a fully factorized local variational distribution (41), the augmented node function ${\tilde{f}}_{b}^{m}\left(s_{b}^{m}\right)$ of (32) reduces to

(A31) $$\begin{array}{cc} {\tilde{f}}_{j}\left(s_{j}\right) & =\exp\left(\int \left\{\prod_{\begin{array}{c} i\in E(b)\\ i\neq j \end{array}}q_{i}\left(s_{i}\right)\right\}\log f_{b}\left(s_{b}\right)d\;s_{b\setminus j}\right)\;. \end{array}$$

The message of (36) then reduces to

$$\begin{array}{cc} \mu_{jc}\left(s_{j}\right) & \;=\;{\tilde{f}}_{j}\left(s_{j}\right)\;, \end{array}$$

which, after substitution, recovers (43). □

### Appendix D.7. Proof of Lemma 4

**Proof.** When we apply the variation $\epsilon \phi_{b}$ to $q_{b}$ and identify the functional derivative $\delta L_{b}/\delta q_{b}$, we recover the result from Appendix D.1, which leads to a solution of the form (47). □

### Appendix D.8. Proof of Theorem 3

**Proof.** We construct the Lagrangian of (46), which by Lemma 4 leads to a solution of the form (47). Substituting this solution in the constraint of (45) leads to

(A32) $$\begin{array}{cc} \left[\overset{\mu_{bj}\left(s_{j}\right)}{\overset{︷}{\int f_{b}\left(s_{b}\right)\prod_{\begin{array}{c} i\in E(b)\\ i\neq j \end{array}}\mu_{ib}\left(s_{i}\right)d\;s_{b\setminus j}}}\right]\mu_{jb}\left(s_{j}\right) & \;=\;\delta (s_{j}-{\hat{s}}_{j})\;. \end{array}$$

This equation is then satisfied by (50), which proves the theorem. □

### Appendix D.9. Proof of Lemma 5

**Proof.** The proof follows directly from Appendix D.1, with ${\tilde{f}}_{b}\left(s_{b};{\hat{s}}_{b}\right)$ substituted for $f_{b}\left(s_{b}\right)$. □

### Appendix D.10. Proof of Theorem 4

**Proof.** Given the result of Lemma 5, the proof follows Appendix D.3, where Laplace propagation chooses the expansion point to be the fixed point ${\hat{s}}_{b}=\arg\max\;\log q_{b}\left(s_{b}\right)$. For all second-order fixed points of the Laplace iterations, it holds that ${\hat{s}}_{b}$ is a fixed point if and only if it is a local optimum of $q_{b}$. The proof is then concluded by Lemma 1 in [76]. □

### Appendix D.11. Proof of Lemma 6

**Proof.** We note that the Lagrange multiplier $\eta_{jb}$ does not depend on $s_{j}$ because the expectation removes all the functional dependencies on $s_{j}$. Furthermore, the expectations of $T_{j}\left(s_{j}\right)$ have the same dimension as the function $T_{j}\left(s_{j}\right)$. This means that the dimension of $\eta_{jb}$ needs to be compatible with that of $T_{j}\left(s_{j}\right)$ so that we can write the constraint as an inner product. We apply the variation $\epsilon \phi_{b}$ to $q_{b}$ and identify the functional derivative $\delta L_{b}/\delta q_{b}$, as

$$\begin{array}{cc} \frac{dL_{b}\left[q_{b}+\epsilon \phi_{b},f_{b}\right]}{d\epsilon }{|}_{\epsilon =0} & =\int \left(\overset{\delta L_{b}/\delta q_{b}}{\overset{︷}{\log\frac{q_{b}\left(s_{b}\right)}{f_{b}\left(s_{b}\right)}+1+\psi_{b}-\sum_{\begin{array}{c} i\in E(b)\\ i\neq j \end{array}}\lambda_{ib}\left(s_{i}\right)-\eta_{jb}^{⊤}T_{j}\left(s_{j}\right)}}\right)\phi_{b}\left(s_{b}\right)d\;s_{b}\;. \end{array}$$

Setting the functional derivative to zero and identifying $\mu_{ib}\left(s_{i}\right)=\exp\lambda_{ib}\left(s_{i}\right)$ for i≠j and identifying $\mu_{jb}\left(s_{j}\right)=\exp\;\left(\eta_{jb}^{⊤}T_{j}\left(s_{j}\right)\right)$ yields the functional form of the stationary solution as (62). □

### Appendix D.12. Proof of Lemma 7

**Proof.** We follow a similar procedure as in Appendix D.11 and apply the variation $\epsilon \phi_{j}$ to $q_{j}$, which identifies the functional derivative $\delta L_{j}/\delta q_{j}$, as

$$\begin{array}{cc} \frac{dL[q_{j}+\epsilon \phi_{j}]}{d\epsilon }{|}_{\epsilon =0} & =\int \left(\overset{\delta L_{j}/\delta q_{j}}{\overset{︷}{-\log q_{j}\left(s_{j}\right)-1+\psi_{j}+\sum_{a\in V(j)}\eta_{ja}^{⊤}T_{j}\left(s_{j}\right)}}\right)\phi_{j}\left(s_{j}\right)d\;s_{j}\;. \end{array}$$

Setting the functional derivative to zero and following the same procedure as in Appendix D.2 yields (64). □

### Appendix D.13. Proof of Theorem 5

**Proof.** By substituting the stationary solutions given by Lemmas 6 and 7 into the moment-matching constraint (59), we obtain the following condition:

$$\begin{array}{cc} \int T_{j}\left(s_{j}\right)q_{j}\left(s_{j}\right)d\;s_{j} & =\int T_{j}\left(s_{j}\right)q_{b}\left(s_{b}\right)d\;s_{b}\\ \frac{1}{Z_{j}}\int T_{j}\left(s_{j}\right)\exp\;\left({\left[\eta_{jb}+\eta_{jc}\right]}^{⊤}T_{j}\left(s_{j}\right)\right)ds_{j} & =\frac{1}{{\tilde{Z}}_{j}}\int T_{j}\left(s_{j}\right)\overset{\mu_{jb}\left(s_{j}\right)}{\overset{︷}{\exp\;\left(\eta_{jb}^{⊤}T_{j}\left(s_{j}\right)\right)}}\left[\overset{{\tilde{\mu }}_{jc}\left(s_{j}\right)}{\overset{︷}{\int f_{b}\left(s_{b}\right)\prod_{\begin{array}{c} i\in E(b)\\ i\neq j \end{array}}\mu_{ib}\left(s_{i}\right)d\;s_{b\setminus j}}}\right]d\;s_{j}\\ \; & =\int T_{j}\left(s_{j}\right){\tilde{q}}_{j}\left(s_{j}\right)d\;s_{j}\;, \end{array}$$

where we recognize the sum-product message ${\tilde{\mu }}_{jc}\left(s_{j}\right)$, which we multiply by the incoming exponential family message $\mu_{jb}\left(s_{j}\right)$ and normalize to obtain ${\tilde{q}}_{j}\left(s_{j}\right)$. By defining $\eta_{j}=\eta_{jb}+\eta_{jc}$, normalization constants are given by

$$\begin{array}{cc} Z_{j}\left(\eta_{j}\right) & =\int \exp\;\left(\eta_{j}^{⊤}T_{j}\left(s_{j}\right)\right)ds_{j}\\ {\tilde{Z}}_{j} & =\int \exp\left(\eta_{jb}^{⊤}T_{j}\left(s_{j}\right)\right){\tilde{\mu }}_{jc}\left(s_{j}\right)ds_{j}\;. \end{array}$$

Computing the moments allows us to determine the exponential family parameter by solving the following equation [24] (Proposition 3.1)

$$\begin{array}{cc} \nabla_{\eta_{j}}\log Z_{j}\left(\eta_{j}\right) & =\int {\tilde{q}}_{j}\left(s_{j}\right)\;T_{j}\left(s_{j}\right)d\;s_{j}\;. \end{array}$$

Suppose you obtain a solution to this equation denoted by ${\tilde{\eta }}_{j}$, this allows us to approximate the sum-product message ${\tilde{\mu }}_{jc}\left(s_{j}\right)$ by an exponential family message whose parameter is given by

$$\begin{array}{c} \eta_{jc}={\tilde{\eta }}_{j}-\eta_{jb}\;. \end{array}$$

Now let us assume that the fixed points of the sum-product iterations ${\tilde{\mu }}_{jc}^{\left(k\right)}\left(s_{j}\right)={\tilde{\mu }}_{jc}^{\left(k+1\right)}\left(s_{j}\right)$ and the incoming exponential family messages $\mu_{jb}^{\left(k\right)}\left(s_{j}\right)=\mu_{jb}^{\left(k+1\right)}\left(s_{j}\right)$ exist for some *k*. Then, we need to show that the existence of these fixed points implies the existence of the fixed points of $\mu_{jc}^{\left(k+1\right)}=\mu_{jc}^{\left(k\right)}$. By moment-matching, we have

$$\begin{array}{cc} \eta_{jc}^{\left(k+1\right)} & ={\tilde{\eta }}_{j}^{\left(k+1\right)}-\eta_{jb}^{\left(k+1\right)}\\ \; & ={\tilde{\eta }}_{j}^{\left(k\right)}-\eta_{jb}^{\left(k\right)}\\ \; & =\eta_{jc}^{\left(k\right)}\;, \end{array}$$

which proves the existence of the fixed point of $\mu_{jc}$ if ${\tilde{\mu }}_{jc}$ and $\mu_{jb}\left(s_{j}\right)$ have fixed points. □

### Appendix D.14. Proof of Theorem 6

**Proof.** The proof follows directly from substituting the Laplace-approximated factor-function (53) in the naive mean-field result of Corollary 1. □

### Appendix D.15. Proof of Theorem 7

**Proof.** In order to obtain the optimal parameter value $\theta_{j}^{*}$, we view the free energy as a function of $\theta_{j}$. As there are two node-local free energies that depend upon $\theta_{j}$, this leads to

$$\begin{array}{cc} \theta_{j}^{*} & =\arg\min_{\theta_{j}}\left(F\left[q_{b},f_{b};\theta_{j}\right]+F\left[q_{c},f_{c};\theta_{j}\right]\right)\\ \; & =\arg\max_{\theta_{j}}\left(\int \left\{\delta \left(s_{j}-\theta_{j}\right)\;\prod_{\begin{array}{c} n\in l(b)\\ n\neq m \end{array}}q_{b}^{n}\left(s_{b}^{n}\right)\right\}\log f_{b}\left(s_{b}\right)d\;s_{b}+\int \left\{\delta \left(s_{j}-\theta_{j}\right)\;\prod_{\begin{array}{c} n\in l(c)\\ n\neq m \end{array}}q_{c}^{n}\left(s_{c}^{n}\right)\right\}\log f_{c}\left(s_{c}\right)d\;s_{c}\right)\\ \; & =\arg\max_{\theta_{j}}\left(\int \left\{\prod_{\begin{array}{c} n\in l(b)\\ n\neq m \end{array}}q_{b}^{n}\left(s_{b}^{n}\right)\right\}\log f_{b}\left(s_{b\setminus j},\theta_{j}\right)d\;s_{b\setminus j}+\int \left\{\prod_{\begin{array}{c} n\in l(c)\\ n\neq m \end{array}}q_{c}^{n}\left(s_{c}^{n}\right)\right\}\log f_{c}\left(s_{c\setminus j},\theta_{j}\right)d\;s_{c\setminus j}\right)\\ \; & =\arg\max_{s_{j}}\left(\log\mu_{bj}\left(s_{j}\right)+\log\mu_{cj}\left(s_{j}\right)\right)\;, \end{array}$$

where in the last step we replaced $\theta_{j}$ with $s_{j}$ for convenience. Here, we recognize $\mu_{bj}$ and $\mu_{cj}$ as the structured variational updates of Theorem 2. Identification of the fixed points can then be obtained by [57] (Corollary 2). For a rigorous discussion on convergence of the EM algorithm, we refer to [77] (Corollary 32), [24] (Chapter 6) and [57] (Section 3). □

### Appendix D.16. Proof of Theorem 8

**Proof.** Substituting for $q_{a}\left(s_{a}\right)$, the node-local free energy becomes

$$\begin{array}{cc} F[q_{a},f_{a}] & =\int q_{a}\left(s_{a}\right)\log\frac{q_{a}\left(s_{a}\right)}{f_{a}\left(s_{a}\right)}d\;s_{a}\\ \; & =\int q_{a}\left(s_{a}\right)\log\frac{q_{j|a}\left(s_{j}|s_{a\setminus j}\right)}{f_{a}\left(s_{a}\right)}d\;s_{a}+\int q_{a}\left(s_{a}\right)\log q_{a\setminus j}\left(s_{a\setminus j}\right)d\;s_{a}\\ \; & =\int q_{a\setminus j}\left(s_{a\setminus j}\right)q_{j|a}\left(s_{j}|s_{a\setminus j}\right)\log\frac{q_{j|a}\left(s_{j}|s_{a\setminus j}\right)}{f_{a}\left(s_{a}\right)}d\;s_{a}+\int q_{a\setminus j}\left(s_{a\setminus j}\right)q_{j|a}\left(s_{j}|s_{a\setminus j}\right)\log q_{a\setminus j}\left(s_{a\setminus j}\right)d\;s_{a}\\ \; & =\int q_{a\setminus j}\left(s_{a\setminus j}\right)\left[\int q_{j|a}\left(s_{j}|s_{a\setminus j}\right)\log\frac{q_{j|a}\left(s_{j}|s_{a\setminus j}\right)}{f_{a}\left(s_{a}\right)}d\;s_{j}\right]d\;s_{a\setminus j}+\int q_{a\setminus j}\left(s_{a\setminus j}\right)\log q_{a\setminus j}\left(s_{a\setminus j}\right)d\;s_{a\setminus j}\\ \; & =E_{q_{a\setminus j}}\;\left[D\;\left[q_{j|a}∥f_{a}\right]\right]-H\left[q_{a\setminus j}\right]\;, \end{array}$$

where the first term expresses an expected Kullback–Leibler divergence, and the second term is a negative entropy. The only possibility for the local free energy to becomes finite, is when $q_{j|a}\left(s_{j}|s_{a\setminus j}\right)=f_{a}\left(s_{a}\right)=\delta \left(s_{j}-g_{a}\left(s_{a\setminus j}\right)\right)$. We then have:

$$\begin{array}{cc} F[q_{a},f_{a}] & =\left\{\begin{array}{cc} -H[q_{a\setminus j}] & \mathrm{if}\;q_{j|a}\left(s_{j}|s_{a\setminus j}\right)=\delta \left(s_{j}-g_{a}\left(s_{a\setminus j}\right)\right)\\ \infty & \mathrm{otherwise}. \end{array}\right. \end{array}$$

□

### Appendix D.17. Proof of Theorem 9

**Proof.** The proof is similar to Appendix D.16. Substituting for $q_{a}\left(s_{a}\right)$, the node-local free energy becomes

$$\begin{array}{cc} F[q_{a},f_{a}] & =\int q_{a}\left(s_{a}\right)\log\frac{q_{a}\left(s_{a}\right)}{f_{a}\left(s_{a}\right)}d\;s_{a}\\ \; & =\int q_{a}\left(s_{i},s_{j},s_{k}\right)\log\frac{q_{ik|j}\left(s_{i},s_{k}|s_{j}\right)}{f_{a}\left(s_{i},s_{j},s_{k}\right)}d\;s_{i}d\;s_{j}d\;s_{k}+\int q_{j}\left(s_{j}\right)\log q_{j}\left(s_{j}\right)d\;s_{j}\\ \; & =E_{q_{j}}\;\left[D\;\left[q_{ik|j}∥f_{a}\right]\right]-H\left[q_{j}\right]\;. \end{array}$$

In contrast to Appendix D.16, here we have a joint belief within the divergence with a single conditioning variable. Conditioning on $s_{j}$ (or by symmetry $s_{i}$ or $s_{k}$) determines the realization of the other variables. Therefore, we have:

$$\begin{array}{cc} F[q_{a},f_{a}] & =\left\{\begin{array}{cc} -H[q_{j}] & \mathrm{if}\;q_{ik|j}\left(s_{i},s_{k}|s_{j}\right)=\delta \left(s_{j}-s_{i}\right)\;\delta \left(s_{j}-s_{k}\right)\\ \infty & \mathrm{otherwise}. \end{array}\right. \end{array}$$

□
